# Ancient DNA from the skeletons of Roopkund Lake reveals Mediterranean migrants in India

**DOI:** 10.1038/s41467-019-11357-9

**Published:** 2019-08-20

**Authors:** Éadaoin Harney, Ayushi Nayak, Nick Patterson, Pramod Joglekar, Veena Mushrif-Tripathy, Swapan Mallick, Nadin Rohland, Jakob Sedig, Nicole Adamski, Rebecca Bernardos, Nasreen Broomandkhoshbacht, Brendan J. Culleton, Matthew Ferry, Thomas K. Harper, Megan Michel, Jonas Oppenheimer, Kristin Stewardson, Zhao Zhang, Harashawaradhana, Maanwendra Singh Bartwal, Sachin Kumar, Subhash Chandra Diyundi, Patrick Roberts, Nicole Boivin, Douglas J. Kennett, Kumarasamy Thangaraj, David Reich, Niraj Rai

**Affiliations:** 1 Department of Organismic and Evolutionary Biology, Harvard University, Cambridge, MA 02138 USA; 2 The Max Planck-Harvard Research Center for the Archaeoscience of the Ancient Mediterranean, Cambridge, MA 02138 USA; 3 Department of Genetics, Harvard Medical School, Boston, MA 02115 USA; 4 Department of Archaeology, Max Planck Institute for the Science of Human History, D-07745 Jena, Germany; 5 Broad Institute of Harvard and MIT, Cambridge, MA 02142 USA USA; 6 Department of Human Evolutionary Biology, Harvard University, Cambridge, MA 02138 USA; 7 Deccan College, Pune, 411006 India; 8 Howard Hughes Medical Institute, Harvard Medical School, Boston, MA 02115 USA; 9 Institutes of Energy and the Environment, The Pennsylvania State University, University Park, PA 16802 USA; 10 Department of Anthropology, The Pennsylvania State University, University Park, PA 16802 USA; 11 The Max Planck-Harvard Research Center for the Archaeoscience of the Ancient Mediterranean, D-07745 Jena, Germany; 12 Anthropological Survey of India, North West Regional Centre, Dehradun, 248195 India; 13 CSIR Centre for Cellular and Molecular Biology, Hyderabad, Telangana 500007 India; 14 Birbal Sahni Institute of Palaeosciences, Lucknow, Uttar Pradesh 226007 India; 15 Gautam Budh Health Care Foundation, Noida, Uttar Pradesh 201301 India; 16 Department of Anthropology, University of California, Santa Barbara, CA 93106 USA

**Keywords:** Genetic variation, Archaeology

## Abstract

Situated at over 5,000 meters above sea level in the Himalayan Mountains, Roopkund Lake is home to the scattered skeletal remains of several hundred individuals of unknown origin. We report genome-wide ancient DNA for 38 skeletons from Roopkund Lake, and find that they cluster into three distinct groups. A group of 23 individuals have ancestry that falls within the range of variation of present-day South Asians. A further 14 have ancestry typical of the eastern Mediterranean. We also identify one individual with Southeast Asian-related ancestry. Radiocarbon dating indicates that these remains were not deposited simultaneously. Instead, all of the individuals with South Asian-related ancestry date to ~800 CE (but with evidence of being deposited in more than one event), while all other individuals date to ~1800 CE. These differences are also reflected in stable isotope measurements, which reveal a distinct dietary profile for the two main groups.

## Abstract (web summary)

Remains of several hundred humans are scattered around Roopkund Lake, situated over 5,000 meters above sea level in the Himalayan Mountains. Here the authors analyze genome-wide data from 38 skeletons and find 3 clusters with different ancestries and dates, showing the people were desposited in multiple catastrophic events.

## Introduction

**Fig. 1:**
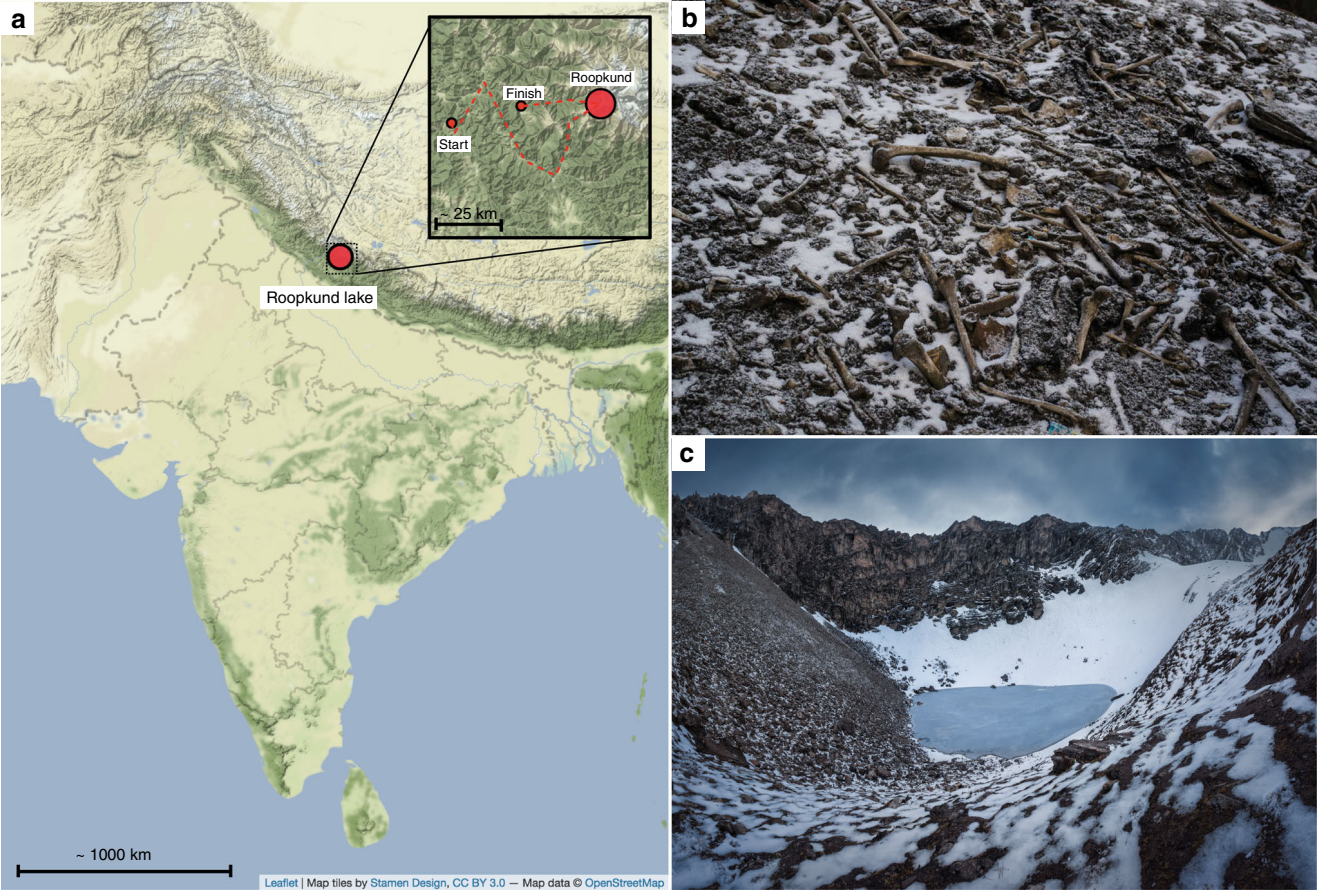
Context of Roopkund Lake. **a** Map showing the location of Roopkund Lake. The approximate route of the Nanda Devi Raj Jat pilgrimage relative to Roopkund Lake is shown in the inset. **b** Image of disarticulated skeletal elements scattered around the Roopkund Lake site. Photo by Himadri Sinha Roy. **c** Image of Roopkund Lake and surrounding mountains. Photo by Atish Waghwase

Nestled deep in the Himalayan mountains at 5029 m above sea level, Roopkund Lake is a small body of water (~40 m in diameter) that is colloquially referred to as Skeleton Lake due to the remains of several hundred ancient humans scattered around its shores (Fig. 1)^1^. Little is known about the origin of these skeletons, as they have never been subjected to systematic anthropological or archaeological scrutiny, in part due to the disturbed nature of the site, which is frequently affected by rockslides^2^, and which is often visited by local pilgrims and hikers who have manipulated the skeletons and removed many of the artifacts^3^. There have been multiple proposals to explain the origins of these skeletons. Local folklore describes a pilgrimage to the nearby shrine of the mountain goddess, Nanda Devi, undertaken by a king and queen and their many attendants, who—due to their inappropriate, celebratory behavior—were struck down by the wrath of Nanda Devi^4^. It has also been suggested that these are the remains of an army or group of merchants who were caught in a storm. Finally, it has been suggested that they were the victims of an epidemic^5^.

To shed light on the origin of the skeletons of Roopkund, we analyzed their remains using a series of bioarcheological analyses, including ancient DNA, stable isotope dietary reconstruction, radiocarbon dating, and osteological analysis. We find that the Roopkund skeletons belong to three genetically distinct groups that were deposited during multiple events, separated in time by approximately 1000 years. These findings refute previous suggestions that the skeletons of Roopkund Lake were deposited in a single catastrophic event.

## Results

### Bioarcheological analysis of the Roopkund skeletons

We obtained genome-wide data from 38 individuals by extracting DNA from powder drilled from long bones, producing next-generation sequencing libraries, and enriching them for approximately 1.2 million single nucleotide polymorphisms (SNPs) from across the genome^6–9^, obtaining an average coverage of 0.51 × at targeted positions (Table 1, Supplementary Data 1). We also obtained PCR-based mitochondrial haplogroup determinations for 71 individuals (35 of these were ones for whom we also obtained genome-wide data that confirmed the PCR-based determinations) (Table 2, Supplementary Note 1). We generated stable isotope measurements (δ^13^C and δ^15^N) from 45 individuals, including 37 for whom we obtained genome-wide genetic data, and we obtained direct radiocarbon dates for 37 individuals for whom we also had both genetic and isotope data (Table 1).

**Table 1:** Information on 38 individuals with genome-wide data

Sample ID	Skeletal codes	No. libraries produced	Population label	Sex	Mitochondrial DNA haplogroup (based on Sequenom genotyping)	Mitochondrial DNA haplogroup (based on mt capture)	Y-chromosome haplogroup	Proportion of endogenous human DNA before capture (best library)	1240k coverage (average)	No. of SNPs hit on autosomes	C-to-T damage rate at terminal bases (average)	X-chromosome contamination point estimate (for males with > 200 SNPs)	Mitochondrial DNA match rate to consensus sequence	Calibrated radiocarbon dates^c^	δ^13^C (‰)^d^	δ^15^N (‰)^d^
I2868	R01	3	Roopkund_A	M	M1a1c	M33d	H1a2a1	0.014	0.868	570995	0.071	..	0.996	890–982 CE	−19.40	−7.69
I2871	R04	4	Roopkund_A	F	M3C1	M3c1a	..	0.005	0.579	441880	0.049	..	0.997	773–940 CE	−16.32	−9.77
I2872	R06	4	Roopkund_A	F	M3c2	M3c2	..	0.003	0.199	196393	0.046	..	1.000	773–940 CE	−19.00	−9.24
I3342	R08	1	Roopkund_A	M	M3a2	M3a2	H1a1d2	0.007	0.577	403739	0.047	0.013	1.000	773–940 CE	−18.94 (−18.88)	−9.69 (−9.85)
I3343	R10	1	Roopkund_A	F	M3	M3	..	0.006	0.223	203058	0.055	..	1.000	773–890 CE	−19.74	−9.99
I3344	R11	1	Roopkund_A	F	U	U2c1	..	0.003	0.105	111184	0.049	..	1.000	775–890 CE	−11.45	−8.71
I3346	R15	1	Roopkund_A	M	..	M30c	E1b1b1	0.004	0.304	271560	0.065	0.008	1.000	717–889 CE	−15.93	−10.29
I3349	R17	1	Roopkund_A	F	..	M5a	..	0.004	0.133	136268	0.059	..	0.998	770–945 CE	−10.74	−9.58
I3351	R19	1	Roopkund_A	M	M3a1	M4	J^b^	0.006	0.044	50278	0.057	..	0.994	770–887 CE	−14.47 (−14.42)	−9.39 (−9.63)
I3352	R20	1	Roopkund_A	M	HV	HV14	R2a3a2b2c	0.017	1.476	591844	0.041	0.004	0.998	689–876 CE	−16.27	−9.13
I3402	R25	1	Roopkund_A	M	M5^a^	U1a1^a^	H3b	0.002	0.118	125762	0.036	..	1.000	770–887 CE	−17.18	−10.36
I3406	R43	1	Roopkund_A	M	M30	M30	J2a1	0.016	0.295	251527	0.045	..	0.999	885–980 CE	−18.46 (−18.07)	7.95 (−8.23)
I3407	R44	1	Roopkund_A	M	M3a1	M3a1	H1a1d2	0.011	0.105	110441	0.045	..	0.976	775–961 CE	−18.22 (−18.27)	−9.85 (−9.69)
I6934	R45	1	Roopkund_A	F	..	..	..	0.034	0.861	521678	0.033	..	1.000	773–890 CE	−16.53	−8.41
I6938	R51	1	Roopkund_A	F	X	X2p	..	0.011	0.481	405124	0.058	..	0.999	694–875 CE	−18.62 (−18.16)	−8.25 (−8.4)
I6941	R55	1	Roopkund_A	M	J1b1a1	J1b1a1	..	0.009	0.590	452228	0.044	−0.001	1.000	894–985 CE	−10.13	−8.90
I6942	R57	1	Roopkund_A	M	P4b1	R30b2a	..	0.008	0.602	470065	0.047	−0.001	1.000	770–887 CE	−18.66 (−18.42)	−8.22 (−8.33)
I6943	R61	1	Roopkund_A	M	M3a1	M3a1	..	0.007	0.133	145489	0.064	..	0.999	675–769 CE	−10.10	−8.24
I6944	R62	1	Roopkund_A	F	U2e3	U4d3	..	0.009	0.340	313369	0.055	..	1.000	726–885 CE	−18.00 (−18.10)	−8.58 (−7.9)
I6945	R64	1	Roopkund_A	F	M4″67	M30 + 16234	..	0.007	0.035	40150	0.045	..	0.997	687–870 CE	−17.08	−8.92
I6946	R65	1	Roopkund_A	M	U2a1	U8b1a1	..	0.005	0.349	328001	0.055	−0.002	1.000	773–890 CE	−10.21	−10.09
I7035	R68	1	Roopkund_A	F	U7	U7a2	..	0.008	0.565	446699	0.041	..	0.999	889–971 CE	−16.74 (−16.50)	−10.19 (−10.21)
I7036	R69	1	Roopkund_A	M	H	H13a2a	..	0.009	0.370	342426	0.057	0.005	1.000	778–988 CE	−18.59	−9.33
I2869	R02	4	Roopkund_B	M	H	H6b1	J1a3a	0.036	0.782	578890	0.057	..	0.997	1668–1945 CE	−18.69	−10.89
I2870	R03	2	Roopkund_B	F	T1	T1a	..	0.024	0.028	31880	0.039	..	0.938	1706–1915 CE	−18.67	−11.15
I3345	R13	1	Roopkund_B	M	H1	H1	R1a1a1b1a2b	0.056	1.547	706651	0.059	0.002	0.997	1681–1939 CE	−18.93	−10.76
I3348	R16	1	Roopkund_B	F	H1	H1c	..	0.006	0.409	352584	0.051	..	1.000	1682–1932 CE	−19.23	−9.21
I3350	R18	1	Roopkund_B	M	H	H60a	G2a2b2a1a1c1a2	0.031	1.349	614489	0.069	0.004	0.995	1675–1943 CE	−19.41 (−19.10)	−9.95 (−10.02)
I3401	R22	1	Roopkund_B	M	N2	W1	R1b1a^b^	0.005	0.049	56291	0.056	..	1.000	..	..	..
I3403	R39	1	Roopkund_B	M	N	X2d	T1a2	0.018	0.492	379935	0.035	0.005	0.995	1691–1925 CE	−18.60 (−18.19)	−10.77 (−10.61)
I3404	R40	1	Roopkund_B	M	H	H12	E1b1b1b2	0.040	1.077	541763	0.041	0.006	0.997	1706–1915 CE	−19.23	−9.62
I3405	R42	1	Roopkund_B	F	J1b	J1b	..	0.019	0.514	346216	0.031	..	1.000	1656-… CE	−19.72	−10.07
I6935	R46	1	Roopkund_B	F	HV	..	..	0.017	0.627	524922	0.060	..	0.997	1668–1945 CE	−18.97	−8.91
I6936	R48	1	Roopkund_B	M	M2a1^a^	H1^b^	..	0.034	1.371	728448	0.043	0.005	0.998	1681–1939 CE	−18.79	−9.79
I6937	R49	1	Roopkund_B	F	H12	H12a	..	0.026	0.837	584656	0.035	..	1.000	1661-… CE	−19.56	−8.93
I6939	R53	1	Roopkund_B	M	H1	H1	..	0.008	0.605	476797	0.037	0.006	0.999	1680–1939 CE	−19.22	−10.46
I6947	R66	1	Roopkund_B	M	K	K1a	..	0.050	0.026	30592	0.025	..	0.940	1675–1943 CE	−18.95	−9.96
I6940	R54	1	Roopkund_C	M	M24	M24a	O1b1a1a1b	0.011	0.489	419098	0.047	0.022	1.000	1653-… CE	−19.25 (−18.32)	−9.98 (−9.74)

^a^Mitochondrial DNA haplogroups that are inconsistent between the capture and PCR-based methods are indicated

^b^Y-chromosome calls that should be interpreted with caution due to low coverage

^c^95.4% confidence interval, rounded to nearest 5 years. Intervals that extend beyond the year 1950 CE are indicated with “..”

^d^Data for 11 individuals generated at the Max Planck Institute for the Science of Human History in Jena are reported in parentheses; the other data were generated at the Yale Analytical and Stable Isotope Center

**Table 2:** Mitochondrial DNA haplogroup determination for 71 individuals

Skeletal codes	mt-DNA haplogroup (determined via multiplex PCR analysis)	Mutational differences from rCRS (determined via multiplex PCR analysis)	Whole-genome ID	mt-DNA haplogroup (determined via whole-genome sequencing)	Population label (determined via whole-genome sequencing)
*R01*	M1a1c	15043, 3384, 7094, 11215	I2868	M33d	Roopkund_A
*R02*	H	2706, 12705, 11719, 14766, 16223	I2869	H6b1	Roopkund_B
*R03*	T1	16294, 16223, 12633, 11251, 15452, 8701, 15607, 1888, 14905, 11215, 9540, 8697, 16126, 12633, 4216, 709	I2870	T1a	Roopkund_B
*R04*	M3C1	15043, 482, 16294	I2871	M3c1a	Roopkund_A
*R05*	M2c	15043, 4216	..	..	..
*R06*	M3c2	15043, 16126, 482	I2872	M3c2	Roopkund_A
*R07*	U4b2	11467, 8701	..	..	..
*R08*	M3a2	15043, 16126, 482, 5783, 10727	I3342	M3a2	Roopkund_A
*R09*	U2b2	1888, 11467, 12308, 2706, 12705, 8701, 1811	..	..	..
*R10*	M3	15043, 16126	I3343	M3	Roopkund_A
*R11*	U	11467, 12308, 8701, 3714, 13188	I3344	U2c1	Roopkund_A
*R12*	M4″67	12007, 15043	..	..	..
*R13*	H1	16223, 14766, 11719, 12705, 9540, 3010, 2706	I3345	H1	Roopkund_B
*R14*	N1b	9540, 8701, 1598	..	..	..
*R15*	..	..	I3346	M30c	Roopkund_A
*R16*	H1	16223, 11719, 5301, 3434, 12705, 9540, 3010, 2706	I3348	H1c	Roopkund_B
*R17*	..	16223, 14766, 11719, 8701, 12705, 9540, 2706	I3349	M5a	Roopkund_A
*R18*	H	15043, 482, 4703	I3350	H60a	Roopkund_B
*R19*	M3a1	9540, 12705, 8701, 11719, 14766, 16223	I3351	M4	Roopkund_A
*R20*	HV	9540, 12705, 8701, 11719, 14766, 16223	I3352	HV14	Roopkund_A
*R21*	HV	709, 16126, 207, 9540, 8701	..	..	..
*R22*	N2	8701, 11719, 14766, 16223	I3401	W1	Roopkund_B
*R23*	HV	15043, 9540, 8701, 12361	..	..	..
*R24*	N1a1b1	1888, 15043, 7094, 7859, 11215, 8701, 16172, 13104, 16223	..	..	..
*R25*	M5^a^	709, 11083, 15043, 8502, 16274, 12810	I3402	U1a1^a^	Roopkund_A
*R26*	M2a	709, 1888, 15043	..	..	..
*R28*	M5	9540, 12705, 8701, 16223	..	..	..
*R29*	R2	15043, 16126, 5301	..	..	..
*R31*	M6	1888, 15043	..	..	..
*R32*	M5	15043	..	..	..
*R33*	M	12007, 15043, 5301, 3714, 13104, 16223, 16294	..	..	..
*R34*	M4″67	1888, 11467, 12308, 2706, 9540, 12705, 8701, 1811	..	..	..
*R35*	U2b	15043	..	..	..
*R36*	M9a2	16126, 9540, 12705, 8701, 1811, 16223	..	..	..
*R37*	HV	11467, 12308, 9540, 12705, 8701, 1811, 16223	..	..	..
*R38*	U2e	6221, 6371, 9540, 8701	..	..	..
*R39*	N	2706, 9540, 12705, 8701, 11719, 14766, 16223	I3403	X2d	Roopkund_B
*R40*	H	2706, 9540, 12705	I3404	H12	Roopkund_B
*R41*	T1	16223, 14766, 11719, 8701, 12705, 9540, 2706, 16126, 15043, 4491	..	..	..
*R42*	J1b	709, 1888, 4216, 12633, 16126, 8697, 9540, 14905, 15607, 8701, 15452, 11251, 12633, 16223	I3405	J1b	Roopkund_B
*R43*	M30	4216, 16126, 3010, 9540, 16612, 12705, 8701, 12406, 15452, 16069, 11251, 16223	I3406	M30	Roopkund_A
*R44*	M3a1	12007, 15043	I3407	M3a1	Roopkund_A
*R45*	..	..	I6934	..	Roopkund_A
*R46*	HV	15043, 16126, 482, 4703	I6935	..	Roopkund_B
*R47*	H	2706, 9540, 12705, 8701, 11719, 14766	..	..	..
*R48*	M2a1^a^	15670, 207, 4703	I6936	H1^a^	Roopkund_B
*R49*	H12	2706, 9540, 12705, 16223	I6937	H12a	Roopkund_B
*R50*	U4	11467, 12308	..	..	..
*R51*	X	6221, 9540, 8701	I6938	X2p	Roopkund_A
*R52*	M6	15043, 5082, 5301	..	..	..
*R53*	H1	2706, 3010, 9540, 12705, 8701, 11719, 14766, 16223	I6939	H1	Roopkund_B
*R54*	M24	15043, 13359, 15607	I6940	M24a	Roopkund_C
*R55*	J1b1a1	4216, 12007, 16126, 3010, 9540, 12612, 12705, 8701, 15452, 16069, 16172, 11251, 16223	I6941	J1b1a1	Roopkund_A
*R56*	M	15043	..	..	..
*R57*	P4b1	12007, 15043	I6942	R30b2a	Roopkund_A
*R59*	D4	15043, 3010, 5178, 8414	..	..	..
*R60*	M4″67	12007, 15043	..	..	..
*R61*	M3a1	15043, 16126, 482, 4703	I6943	M3a1	Roopkund_A
*R62*	U2e3	16223, 1811, 8701, 12705, 9540, 12308, 11467	I6944	U4d3	Roopkund_A
*R63*	U2e3	11467, 12308, 9540, 12705, 8701, 1811, 16223	..	..	..
*R64*	M4″67	12007, 15043	I6945	M30 + 16234	Roopkund_A
*R65*	U2a1	11467, 12308, 9540, 12705, 8701, 10609, 1811, 16223	I6946	U8b1a1	Roopkund_A
*R66*	K	11467, 12308, 8701, 1811, 16223	I6947	K1a	Roopkund_B
*R67*	M	15043	..	..	..
*R68*	U7	11467, 12308, 9540, 12705, 8701, 14569, 1811, 16223	I7035	U7a2	Roopkund_A
*R69*	H	709, 2706, 9540, 12705, 8701, 11719, 14766, 16223	I7036	H13a2a	Roopkund_A
*R72*	T	4216, 16126, 9540, 12705, 8701, 16223	..	..	..
*R73*	U	3741, 12308, 11467	..	..	..
*R74*	U	11467, 12308	..	..	..
*R76*	JT	16126, 12308	..	..	..
*R77*	U	11467, 12308	..	..	..

^a^Denotes cases where mitochondrial DNA haplogroup determination differs substantially between the multiplex-PCR-based method and mitochondrial capture based analysis

In this study, we also present an osteological assessment of health and stature performed on a different set of bones from Roopkund; this report was drafted well before genetic results from Roopkund were available but was never formally published (an edited version of the original report is presented here as Supplementary Note 2). The analysis suggests that the Roopkund individuals were broadly healthy, but also identifies three individuals with unhealed compression fractures; the report hypothesizes that these injuries could have transpired during a violent hailstorm of the type that sometimes occurs in the vicinity of Roopkund Lake, while also recognizing that other scenarios are plausible. The report also identifies the presence of both very robust and tall individuals (outside the range of almost all South Asians), and more gracile individuals, and hypothesizes based on this the presence of at least two distinct groups of individuals, consistent with our genetic findings (Supplementary Note 2).

Our analysis of the genome-wide data from 38 Roopkund individuals shows that they include both genetic males (*n* = 23) and females (*n* = 15)—consistent with the physical anthropology evidence for the presence of both males and females (Supplementary Note 2). The relatively similar proportions of males and females is difficult to reconcile with the suggestion that these individuals might have been part of a military expedition. We detected no relative pairs (3rd degree or closer) among the sequenced individuals^10^, providing evidence against the idea that the Roopkund skeletons might represent the remains of groups of families. We also found no evidence that the individuals were infected with bacterial pathogens, providing no support for the suggestion that these individuals died in an epidemic, although we caution that failure to find evidence for pathogen DNA in long bone powder may simply reflect the fact that it was present at too low a concentration to detect (Supplementary Note 3)^11^.

### Roopkund skeletons form three genetically distinct groups

**Fig. 2:**
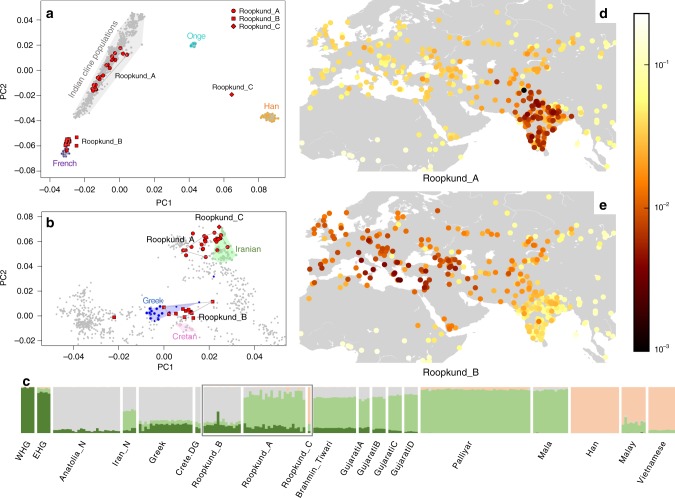
Genetic Structure of the Skeletons of Roopkund Lake. **a** Principal component analysis (PCA) of 1,453 present day individuals from selected groups throughout mainland South Asia (highlighted in gray). French individuals (representing the location where West Eurasian populations are known to cluster) are shown in purple, Chinese individuals are shown (representing the location where East Asian populations are known to cluster) in orange, and Andamanese individuals are shown in teal; the 38 Roopkund individuals are projected. **b** PCA of 988 present day West Eurasians with the Roopkund individuals projected. The PCA plot is truncated to remove Sardinians and southern Levantine groups; Present-day Greeks are shown in blue, Cretans in pink, Iranians in green, and all other West Eurasian populations in gray. A gray polygon encloses all the individuals in each Roopkund group with > 100,000 SNPs. **c** ADMIXTURE analysis of 2344 present-day and 1877 ancient individuals with *K* = 4 ancestral components. Only a subset of individuals with ancestries relevant to the interpretation of the Roopkund individuals are shown. Consistent with the PCA, Roopkund_A has ancestry most closely matching Indian groups; Roopkund_B has ancestry most closely matching Greek and Cretan groups; and Roopkund_C has ancestry most closely matching Southeast Asian groups. Genetic differentiation (F_ST_) between Roopkund_A (**d**) and diverse present-day populations, and Roopkund_B (**e**) and diverse present-day populations. We only plotted present-day populations for which we have latitudes and longitudes; deeper red coloration indicates less differentiation to the Roopkund genetic cluster being analyzed. The plotted data are provided in a Source Data file

We explored the genetic diversity of the 38 Roopkund individuals using a previously established Principal Component Analysis (PCA) that is effective at visualizing genetic variation of diverse present-day people from South Asia (a term we use to refer to the territories of the present day countries of India, Pakistan, Nepal, Bhutan, Bangladesh, and Bhutan) relative to West Eurasian-related groups (a term we use to refer to the cluster of ancestry types common in Europe, the Near East, and Iran) and East Asian-related groups (a term we apply to the cluster of ancestry types common in East Asia including China, Japan, Southeast Asia, and western Indonesia)^12^. We find that the Roopkund individuals cluster into three distinct groups, which we will henceforth refer to as Roopkund_A, Roopkund_B, and Roopkund_C (Fig. 2a). Individuals in Roopkund_A (*n* = 23) fall along a genetic gradient that includes most present-day South Asians. However, they do not fall in a tight cluster along this gradient, suggesting that they do not comprise a single endogamous group, and instead derive from a diversity of groups. Individuals belonging to the Roopkund_B cluster (*n* = 14) do not fall along this gradient, and instead fall near present-day West Eurasians, suggesting that Roopkund_B individuals possess West Eurasian-related ancestry. A single individual, Roopkund_C, falls far from all other Roopkund individuals in the PCA, between the Onge (Andaman Islands) and Han Chinese, suggesting East Asian-related ancestry.

To further understand the West Eurasian-related affinity in the Roopkund_B cluster, we projected all the Roopkund individuals onto a second PCA designed to distinguish between sub-components of West Eurasian-related ancestry^13,14^ (Fig. 2b). Individuals assigned to the Roopkund_A and Roopkund_C groups cluster towards the top right of the PCA plot, close to present-day groups with Iranian ancestry, consistent with where populations with South Asian or East Asian ancestry cluster when projected onto such a plot^13^. Individuals belonging to the Roopkund_B group cluster toward the center of the plot, close to present-day people from mainland Greece and Crete^15^. We observe consistent patterns using the automated clustering software ADMIXTURE^16^ (Fig. 2c) and in pairwise F_ST_ statistics (Fig. 2d, e, Supplementary Data 2). The visual evidence from the PCA suggests that two individuals from the Roopkund_B group might represent genetic outliers (Fig. 2b). However, symmetry *f*_4_-statistics show that the two apparent outliers (one of which has relatively low coverage) are statistically indistinguishable in ancestry from individuals of the main Roopkund_B cluster relative to diverse comparison populations (Supplementary Data 3), and so we lump all the Roopkund_B individuals together in what follows.

### Skeletons at Roopkund Lake were deposited in multiple events

**Fig. 3:**
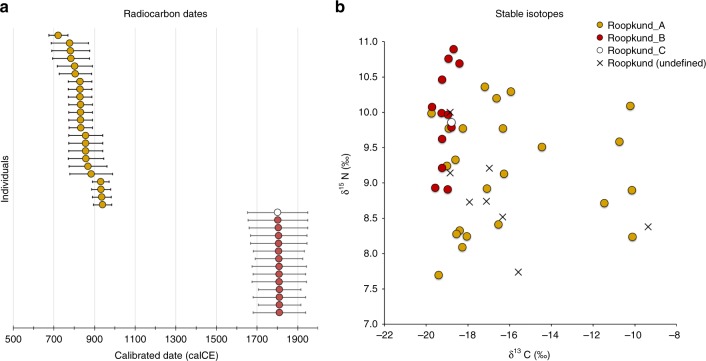
Radiocarbon and Isotopic Evidence of Distinct Origins of Roopkund Genetic Groups. **a** We generated 37 accelerator mass spectrometry radiocarbon dates and calibrated them using OxCal v4.3.2. The dating reveals that the individuals were deposited in at least two events ~1000 years apart. In fact, the Roopkund_A individuals (shown in yellow) may have been deposited over an extended period themselves, as the 95% confidence intervals for some of the radiocarbon dates (for example I6943 and I6941) do not overlap. Radiocarbon dates indicate that Roopkund_B (shown in red) and Roopkund_C (shown in white) individuals may have been deposited during a single event. Error bars indicate 95.4% confidence intervals. Calibration curves are shown in Supplementary Fig. 1. **b** We show normalized δ^13^C and δ^15^N values for samples with isotopic data: 37 for which genetic data were generated (circles with colors indicating their cluster), and eight for which no genetic data were generated (labeled Roopkund_U). In cases where multiple measurements were obtained, we plot the average of all measurements. The plotted data are provided as a Source Data file

The discovery of multiple, genetically distinct groups among the skeletons of Roopkund Lake raises the question of whether these individuals died simultaneously or during separate events. We used Accelerator Mass Spectrometry (AMS) radiocarbon dating to determine the age of the remains. We successfully generated radiocarbon dates from all but one of the individuals for which we have genetic data, using the same stocks of bone powder that we used for genetic analysis to ensure that the dates correspond directly to the genetic groupings. We find that the Roopkund_A and Roopkund_B groups are separated in time by ~1000 years, with the calibrated dates for individuals assigned to the Roopkund_A group ranging from the 7th–10th centuries CE, and the calibrated dates for individuals assigned to the Roopkund_B group ranging from the 17th–20th centuries CE (Table 1; Fig. 3a; Supplementary Data 4). The single individual assigned to Roopkund_C also dates to this later period. These results demonstrate that the skeletons of Roopkund Lake perished in at least two separate events. For Roopkund_A, we detect non-overlapping 95% confidence intervals (for example individual I6943 dates to 675–769 CE, while individual I6941 dates to 894–985 CE), suggesting that even these individuals may not have died simultaneously (Fig. 3a). In contrast, the calibrated dates obtained for 13 Roopkund_B individuals and the single Roopkund_C individual all have mutually overlapping 95% confidence intervals.

### Differences in diet correlate with genetic groupings

We carried out carbon and nitrogen isotope analysis of femur bone collagen for 45 individuals. Femur bone collagen is determined by diet in the last 10–20 years of life^17^, and therefore is not necessarily correlated with the genetic ancestry of a population, which reflects processes occurring over generations. Nevertheless, we find evidence of dietary heterogeneity across the genetic ancestry groupings, providing additional support for the presence of multiple distinct groups at Roopkund Lake. We first observed that the Roopkund individuals are characterized by a range of δ^13^C values indicating diets reliant on both C_3_ and C_4_ plant sources, as well as δ^15^N values indicating varying degrees of consumption of protein derived from terrestrial animals (Fig. 3b and Supplementary Note 4). The δ^13^C values are non-randomly associated with the genetic groupings for the 37 individuals for whom we had both measurements. We find that all the Roopkund_B individuals (with typically eastern Mediterranean ancestry), as well as the Roopkund_C individual, have δ^13^C values between −19.7‰ and −18.2‰ reflecting consumption of terrestrial C_3_ plants, such as wheat, barley, and rice (and/or animals foddered on such plants). In contrast, the Roopkund_A individuals (with typically South Asian ancestry) have much more varied δ^13^C values (−18.9‰ to −10.1‰), with some implying C_3_ plant reliance and others reflecting either a mixed C_3_ and C_4_ derived diet, or alternatively consumption of C_3_ plants along with animals foddered with millet, a C_4_ plant (a practice that has been documented ethnographically in South Asia^17^). The difference in the δ^13^C distribution between the Roopkund_A and Roopkund_B groupings is highly significant (p = 0.00022 from a two-sided Mann-Whitney test).

### Genetic affinities of the Roopkund subgroups

We used *qpWave*^18,19^ to test whether Roopkund_B is consistent with forming a genetic clade with any present-day population (that is, whether it is possible to model the two populations as descending entirely from the same ancestral population with no mixture with other groups since their split). We selected 26 present-day populations for comparison, with particular emphasis on West Eurasian-related groups (we analyzed the West Eurasian-related groups Basque, Crete, Cypriot, Egyptian, English, Estonian, Finnish, French, Georgian, German, Greek, Hungarian, Italian_North, Italian_South, Norwegian, Spanish, Syrian, Ukranian, and the non-West-Eurasian-related groups Brahmin_Tiwari, Chukchi, Han, Karitiana, Mala, Mbuti, Onge, and Papuan). We find that Roopkund_B is consistent with forming a genetic clade only with individuals from present-day Crete. These results by no means imply that the Roopkund_B individuals originated in the island of Crete itself, although they suggest that their recent ancestors or they themselves came from a nearby region (Supplementary Note 5; Supplementary Data 5).

We performed a similar analysis on individuals belonging to the Roopkund_A group and find that they cannot be modeled as deriving from a homogeneous group (Supplementary Note 6). Instead, Roopkund_A individuals vary significantly in their relationship to a diverse set of present-day South Asians, consistent with the heterogeneity evident in PCA (Fig. 2a). We were unable to model the Roopkund_C individual as a genetic clade with any present-day populations, but we were able to model its ancestry as ~82% Malay-related and ~18% Vietnamese-related using *qpAdm*^7^, showing that this individual is consistent with being of Southeast Asian origin. We tested if any of the Roopkund groups show specific genetic affinity to present-day groups from the Himalayan region, including four neighboring villages in the northern Ladakh region for which we report new genome-wide sequence data, but we find no such evidence (Supplementary Note 7). Within the Roopkund_A group which has ancestry that falls within the variation of present-day South Asians, we observe a weakly significant difference in the proportion of West Eurasian-related ancestry in males and females (*p* = 0.015 by a permutation test across individuals; Supplementary Note 8), with systematically lower proportions of West Eurasian-related ancestry in males than females. This suggests that the males and females were drawn from significantly different mixtures of groups within South Asia.

## Discussion

The genetically, temporally, and isotopically heterogeneous composition of the groups at Roopkund Lake was unanticipated from the context in which the skeletons were found. Radiocarbon dating reveals at least two key phases of deposition of human remains separated by around one thousand years and with significant heterogeneity in the dates for the earlier individuals indicating that they could not all have died in a single catastrophic event.

Combining multiple lines of evidence, we suggest a possible explanation for the origin of at least some of the Roopkund_A individuals. Roopkund Lake is not situated on any major trade route, but it is on a present-day pilgrimage route—the Nanda Devi Raj Jat pilgrimage which today occurs every 12 years (Fig. 1a). As part of the event, pilgrims gather for worship and celebration along the route. Reliable descriptions of the pilgrimage ritual do not appear until the late-19^th^ century, but inscriptions in nearby temples dating to between the 8^th^ and 10^th^ centuries suggest potential earlier origins^20^. We view the hypothesis of a mass death during a pilgrimage event as a plausible explanation for at least some of the individuals in the Roopkund_A cluster.

The Roopkund_B cluster is more puzzling. It is tempting to hypothesize that the Roopkund_B individuals descend from Indo-Greek populations established after the time of Alexander the Great, who may have contributed ancestry to some present-day groups like the Kalash^21^. However, this is unlikely, as such a group would be expected to have admixture with groups with more typical South Asian ancestry (as the Kalash do), or would be expected to be inbred and to have relatively low genetic diversity. However, the Roopkund_B individuals have evidence for neither pattern (Supplementary Note 9). Combining different lines of evidence, the data suggest instead that what we have sampled is a group of unrelated men and women who were born in the eastern Mediterranean during the period of Ottoman political control. As suggested by their consumption of a predominantly terrestrial, rather than marine-based diet, they may have lived in an inland location, eventually traveling to and dying in the Himalayas. Whether they were participating in a pilgrimage, or were drawn to Roopkund Lake for other reasons, is a mystery. It would be surprising for a Hindu pilgrimage to be practiced by a large group of travelers from the eastern Mediterranean where Hindu practices have not been common; Hindu practice in this time might be more plausible for a southeast Asian individual with an ancestry type like that seen in the Roopkund_C individual. Given that the Roopkund_B and Roopkund_C individuals died only in the last few centuries, an important direction for future investigation will be to carry out archival research to determine if there were reports of large foreign traveling parties dying in the region over the last few hundred years.

Taken together, these results have produced meaningful insights about an enigmatic ancient site. More generally, this study highlights the power of biomolecular analyses to obtain rich information about the human story behind archaeological deposits that are so highly disturbed that traditional archaeological methods are not as informative.

## Methods

The genetic analysis of Himalayan populations (described in Supplementary Note 7) was approved by the Institutional Ethical Committee of the Centre for Cellular and Molecular Biology in Hyderabad, India.

### Ancient DNA laboratory Work

A total of 76 skeletal samples (72 long bones and four teeth) were sampled at the Anthropological Survey of India, Kolkata. Skeletal sampling was performed for all samples in dedicated ancient DNA facilities at the Centre for Cellular and Molecular Biology (CCMB) in Hyderabad, India. A subset of samples that underwent preliminary ancient DNA screening at CCMB, including three samples that did not yield sufficient data to assign mitochondrial DNA haplogroups during preliminary screening (see Supplementary Note 1), were further processed at Harvard Medical School, Boston, USA, consistent with recommendations in the ancient DNA literature for repeating analyses in two independent laboratories to increase confidence in results^22^.

At CCMB, samples were prepared for processing by wiping with a bleach solution, followed by deionized water. The samples were then subjected to UV irradiation for 30 min on each side to minimize surface DNA contamination. Bone powder was then produced using a sterile dentistry drill.

We successfully generated genome-wide DNA for 38 individuals (Supplementary Data 1). For each sample, approximately 75 mg of bone powder originally prepared at CCMB was further processed in dedicated ancient DNA clean rooms at Harvard Medical School using standard protocols, including DNA extraction optimized for ancient DNA recovery^23^, modified by replacing the Zymo extender/MinElute column assemblage with a preassembled spin column device^24^, followed by library preparation with partial UDG treatment^25^. The quality of authentic ancient DNA preservation in each sample was assessed by carrying out a preliminary screening of all libraries via targeted DNA enrichment, designed to capture mitochondrial DNA in addition to 50 nuclear targets^26^. We sequenced the enriched libraries on an Illumina NextSeq500 instrument for 2 × 76 cycles with an additional 2 × 7 cycles for identification of indices. Based on this preliminary assessment, libraries that were deemed promising underwent a further enrichment using a reagent that targeted ~1.2 million SNPs^6–9^, and then were sequenced using an Illumina NextSeq500 instrument.

### Bioinformatic processing

We used SeqPrep to trim adapters and molecular barcodes, and then merged paired-end reads that overlapped by a minimum of 15 base pairs (with up to one mismatch allowed) and aligned to the mitochondrial *rsrs* genome^27^ (for the mitochondrial screening analysis) or *hg19* (for whole-genome analysis) using *samse* in *bwa* (v0.6.1)^28^. We identified duplicate sequences based on having the same start position, end position, orientation, and library-specific barcode, and only retained the copy with the highest quality sequence. We restricted to sequences with a minimum mapping quality (MAPQ ≥ 10) and minimum base quality (≥20) after excluding two bases from each end of the sequence. We obtained pseudo-haploid SNP calls by using a single randomly chosen sequence at SNPs covered by at least one sequence.

We subjected the resulting data to three tests of ancient DNA authenticity: (1) we analyzed the mitochondrial genome data to determine the rate of matching to the consensus sequence using contamMix, and excluded from analysis samples that exhibited a match rate less than 97%^8^. (2) We removed samples that exhibited a rate of C-to-T substitutions less than 3%: the minimum recommended threshold for authentic ancient DNA that has been subjected to partial UDG treatment^25^. (3) We used ANGSD^29^ to determine the degree of heterogeneity on the X-chromosome in males (who should only have one X chromosome) and excluded from analysis individuals with contamination rates greater than 1.5%.

We determined the mitochondrial haplogroup of each individual in two ways. For individuals with whole mitochondrial genome data, we determined the mitochondrial haplogroups using *haplogrep2*^30^. We also determined mitochondrial haplogroups from mitochondrial DNA genotyping using multiplex PCR (see Supplementary Note 1).

We determined the genetic sex of the individuals by computing the ratio of the number of sequences that align to the X chromosome versus the Y chromosome. We searched for 1st, 2nd, and 3rd degree relative pairs in the dataset by analyzing patterns of allele sharing between pairs of individuals (we found none)^10^.

To identify Y-chromosome haplogroups in genetically male individuals, we used a modified version of the procedure reported in Poznik, et al.^31^, which performs a breadth-first search of the Y-chromosome tree. We made Y chromosome haplogroup calls using the ISOGG tree from 04.01.2016 [http://isogg.org], and recorded the derived and ancestral allele calls for each informative position on the tree. We counted the number of mismatches in the observed derived alleles on each branch of the tree and used this information to assign a score to each haplogroup, accounting for damage by down-weighting derived mutations that are the result of transitions to 1/3 of that of transversions. We assigned the closest matching Y-chromosome reference haplogroup to each male based on this score (Supplementary Data 6). We caution that males with fewer than 100,000 SNPs have too little data to confidently assign a haplogroup.

### Population genetic analyses

We report data for 38 samples that passed contamination and quality control tests, with an average coverage of 0.51 × [range: 0.026–1.547] and 350088 SNPS covered at least once [range 30592–728448]. We processed the data in conjunction with published DNA obtained from ancient^6,9,13–15,32–61^ and present-day groups from throughout the world^62–68^, including ~175 modern groups from the Indian subcontinent^12^. The resulting merged dataset included 1521 ancient and 7985 present-day individuals at 591,304 SNPs.

We used *smartpca*^69^ to perform principal component analysis (PCA) using default parameters, with the settings lsqproject:YES and numoutlier:0. We projected the Roopkund individuals onto two PCA plots designed either to reveal a cline of West Eurasian-related ancestry in South Asian populations^18^, or to reveal the genetic substructure in present-day West Eurasians^13^. The first PCA (Fig. 2a) included 1453 present-day populations^12^ in addition to the Roopkund individuals, while the second PCA (Fig. 2b) included 986 present-day populations^13^, in addition to the Roopkund individuals and two individuals from present-day Crete (population label Crete.DG). The PCA plots show that the samples cluster into three distinct groups, which we label Roopkund_A, Roopkund_B and Roopkund_C, and treat separately for subsequent analyses.

We used *smartpca*^69^ to compute F_ST_ between the two major Roopkund groups (Roopkund_A and Roopkund_B) and all other groups composed of at least 2 individuals in the dataset, using default parameters, with the settings inbreed:YES and fstonly:YES.

We performed clustering using ADMIXTURE^16^. We carried out this analysis on all samples used for the PCA analyses, although we display only selected populations for the sake of clarity. Prior to analysis, SNPs in linkage disequilibrium with one another were pruned in PLINK using the parameters–indep-pairwise 200 25 0.4. We performed an ADMIXTURE analysis on the remaining 344,363 SNPs in the pruned dataset for values of k between 2 and 10, and carried out 20 replicates at each value of *k*. We retained the highest likelihood replicate at each *k* and displayed results for *k* (k = 4), which we chose because we observed that it is most visually helpful for discriminating the ancestry of the groups of interest.

We used *qpWave*^18,19^, with default parameters and allsnps:YES, to determine if any of the Roopkund populations was consistent with being a clade with any present-day populations. We included a base set of nine populations in each test, chosen to represent diverse ancestry from throughout the world. We include an additional 5–15 populations of either South Asian, West Eurasian, or Southeast/East Asian ancestry in tests involving Roopkund_A, Roopkund_B and Roopkund_C respectively, chosen to provide additional resolution for each group based on their position in the previous PCA. Based on the observed genetic heterogeneity in the Roopkund_A population, we modeled each individual separately (Supplementary Note 6). For each test, the Left population set included the Roopkund population or individual of interest in addition to one of the selected present-day analysis populations, while the remaining populations were included in the Right population set. In the case of individuals belonging to the Roopkund_A and Roopkund_C groups, we also used *qpAdm*^7^, with default parameters and allsnps: YES, to determine whether these populations could be considered to be the product of a two-way admixture between any of the selected present-day populations (Supplementary Note 6). In this case, the Left population set included the Roopkund individual of interest in addition to all possible combinations of two of the selected present-day analysis populations, while the remaining populations were included in the Right population set.

### AMS radiocarbon dating

We subjected bone powder from 37 samples to radiocarbon dating. We dated the remaining bone powder (360–750 mg) from the same samples that were processed for ancient DNA. We were unable to generate a radiocarbon date for individual I3401, as there was not enough remaining bone powder for analysis.

At the Pennsylvania State University AMS radiocarbon dating facility, bone collagen for ^14^C and stable isotope analyses was extracted and purified using a modified Longin method with ultrafiltration^70^. Samples (200–400 mg) were demineralized for 24–36 h in 0.5 N HCl at 5 °C followed by a brief (<1 h) alkali bath in 0.1 N NaOH at room temperature to remove humates. The residue was rinsed to neutrality in multiple changes of Nanopure H_2_O, and then gelatinized for 12 h at 60 °C in 0.01 N HCl. The resulting gelatin was lyophilized and weighed to determine percent yield as a first evaluation of the degree of bone collagen preservation. Rehydrated gelatin solution was pipetted into pre-cleaned Centriprep^71^ ultrafilters (retaining >30 kDa molecular weight gelatin) and centrifuged 3 times for 20 min, diluted with Nanopure H_2_O and centrifuged 3 more times for 20 min to desalt the solution.

In some instances, collagen samples were too poorly preserved and were pre-treated at Penn State using a modified XAD process^72^ (Supplementary Data 4 shows that there were no systematic differences in the dates obtained based on the XAD and modified Longin pretreatment extraction methods.) Samples were demineralized in 0.5 N HCl for 2–3 days at 5 °C. The demineralized collagen pseudomorph was gelatinized at 60 °C in 1–2 mL 0.01 N HCl for 8–10 h. The gelatin was then lyophilized and percent gelatinization and yield determined by weight. The sample gelatin was then hydrolyzed in 2 mL 6 N HCl for 24 h at 110 °C. Supelco ENVI-Chrom® SPE (Solid Phase Extraction; Sigma-Aldrich) columns were prepped with 2 washes of methanol (2 mL) and rinsed with 10 mL DI H_2_O. Supelco ENVIChrom® SPE (Solid Phase Extraction; Sigma-Aldrich) columns with 0.45 µm Millex Durapore filters attached were equilibrated with 50 mL 6 N HCl and the washings discarded. 2 mL collagen hydrolyzate as HCl was pipetted onto the SPE column and driven with an additional 10 mL 6 N HCl dropwise with the syringe into a 20 mm culture tube. The hydrolyzate was finally dried into a viscous syrup by passing UHP N_2_ gas over the sample heated at 50 °C for ~12 h.

For all bone samples that were subject to radiocarbon dating, carbon and nitrogen concentrations and stable isotope ratios of the ultrafiltered gelatin or XAD amino acid hydrolyzate were measured at the Yale Analytical and Stable Isotope Center with a Costech elemental analyzer (ECS 4010) and Thermo DeltaPlus analyzer. Sample quality was evaluated by percentage crude gelatin yield, %C, %N, and C/N ratios before AMS ^14^C dating. C/N ratios for all samples fell between 2.9 and 3.6, indicating good collagen preservation^73^. Samples (~2.1 mg) were then combusted for 3 h at 900 °C in vacuum-sealed quartz tubes with CuO and Ag wires. Sample CO_2_ was reduced to graphite at 550 °C using H_2_ and a Fe catalyst, with reaction water drawn off with Mg(ClO_4_)_2_^74^.

Graphite samples were pressed into targets in Al boats and loaded on a target wheel with OX-1 (oxalic acid) standards, known-age bone secondaries, and a ^14^C-free Pleistocene whale blank. ^14^C measurements were performed at UCIAMS on a modified National Electronics Corporation compact spectrometer with a 0.5 MV accelerator (NEC 1.5SDH-1). The ^14^C ages were corrected for mass-dependent fractionation with δ^13^C values^75^ and compared with samples of Pleistocene whale bone (backgrounds, 48,000 ^14^C BP), late Holocene bison bone (~1850 ^14^C BP), late 1800s CE cow bone and OX-2 oxalic acid standards for calibration. All calibrated ^14^C ages were computed using OxCal version 4.3^76^ using the IntCal13 northern hemisphere curve^77^.

### Stable isotope measurements

The isotopic measurement procedure at Yale University for the 37 samples for which we performed direct radiocarbon dating are described in the previous section.

We also obtained isotopic measurements for long bone samples from 19 individuals (including data from 11 of the same individuals that were also analyzed at Yale) at the Max Planck Institute for the Science of Human History. Bone samples of 1 g were subsequently cleaned using an air abrasive system with 5 μm aluminum oxide powder and then crushed into chunks. Collagen was extracted following standard procedures^78^. Approximately 1 g of pre-cleaned bone was demineralized in 10 mL aliquots of 0.5 M HCl at 4 °C, with changes of acid until CO_2_ stopped evolving. The residue was then rinsed three times in deionized water before being gelatinized in pH 3 HCl at 80 °C for 48 h. The resulting solution was filtered, with the supernatant then freeze-dried over a period of 24 h.

Purified collagen samples (1 mg) were analyzed at the Department of Archaeology, Max Planck Institute for the Science of Human History, in duplicate by EA-IRMS on a ThermoFisher Elemental Analyzer coupled to a ThermoFisher Delta V Advantage Mass Spectrometer via a ConFloIV system. Accuracy was determined by measurements of international standard reference materials within each analytical run. These were USGS 40,40 δ^13^C_raw_ = −26.4 ± 0.1, δ^13^C_true_ = −26.4 ± 0.0, δ^15^N_raw_ = −4.4 ± 0.1, δ^15^N_true_ = −4.5 ± 0.2; IAEA N2, δ^15^N_raw_ = 20.2 ± 0.1, δ^15^N_true_ = 20.3 ± 0.2; IAEA C6 δ^13^C_raw_ = -10.9 ± 0.1, δ^13^C_true_ = −10.8 ± 0.0. An in-house fish gelatin sample was also used as a standard in each run. Reported δ^13^C values were measured against Vienna Pee Dee Belemnite (VPDB), while δ^15^N values are measured against ambient air.

### Reporting summary

Further information on research design is available in the Nature Research Reporting Summary linked to this article.

## Supplementary information

Supplementary Information

Peer Review File

Reporting Summary

Description of Additional Supplementary Files

Supplementary Data 1

Supplementary Data 2

Supplementary Data 3

Supplementary Data 4

Supplementary Data 5

Supplementary Data 6

Supplementary Data 7

Supplementary Data 8

Supplementary Data 9

Supplementary Data 10

Supplementary Data 11

Supplementary Data 12

Supplementary Data 13

Supplementary Data 14

Supplementary Data 15

Supplementary Data 16

Supplementary Data 17

Supplementary Data 18

Supplementary Data 19

Supplementary Data 20

Source Data

## Data Availability

The aligned DNA sequences from the 38 individuals are available from the European Nucleotide Archive under accession number PRJEB29537. Genotype files are available at https://reich.hms.harvard.edu/datasets. All other relevant data is available upon request.
