# Supplementary material for: Ancient DNA from the skeletons of Roopkund Lake reveals Mediterranean migrants in India
Source: Nat Commun. 2019 Aug 20;10:3670. doi: 10.1038/s41467-019-11357-9 (PMC6702210; doi:10.1038/s41467-019-11357-9)
Supplement: Supplementary file 1 — Supplementary Information [file 41467_2019_11357_MOESM1_ESM.pdf]

Supplementary Information

Harney et al.

Ancient DNA from the skeletons of Roopkund Lake reveals Mediterranean migrants in South Asia

Table of Contents

Supplementary Figure 1. Radiocarbon Dating Calibration Curve. 2

Supplementary Note 1- Mitochondrial Haplogroup Determination via Multiplex PCR............ 3

Supplementary Note 2- Physical Anthropology Assessment of Roopkund Skeletons............... 5

Supplementary Note 3- Genetic screening for ancient pathogens 13

Supplementary Note 4- Stable carbon and nitrogen isotope analysis of bone collagen 17

Supplementary Note 5- Relationship of Roopkund_B to individuals from present-day Crete 19

Supplementary Note 6- Modeling the ancestry of Roopkund_A and Roopkund_C individuals22

Supplementary Note 7 - No affinity of Roopkund_A to modern Himalayan groups 25

Supplementary Note 8- Systematic ancestry differences in Roopkund_A males and females 29

Supplementary Note 9- Constraints on the origin of Roopkund_B.. 33

References.. 36

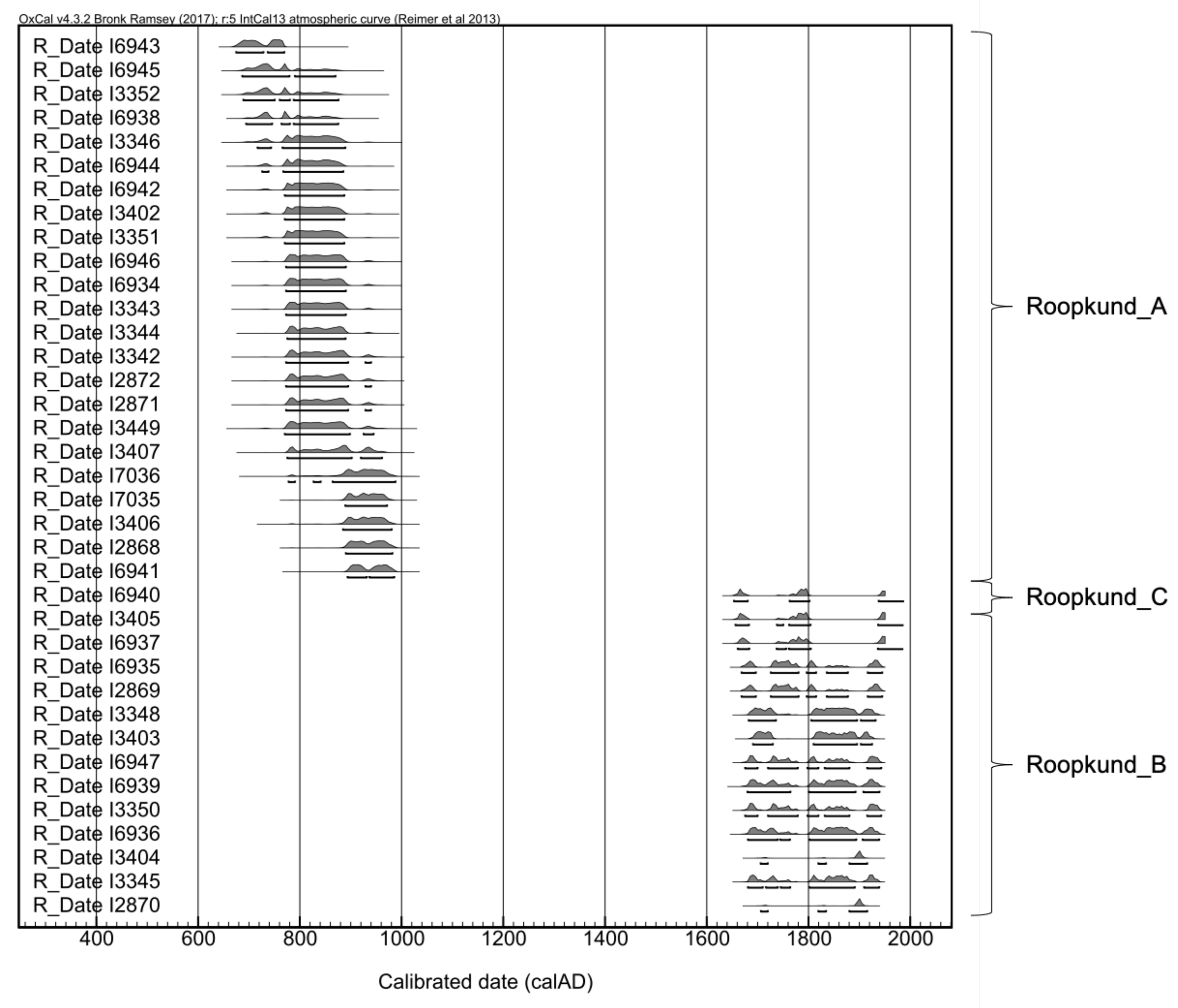

Supplementary Figure 1. Radiocarbon Dating Calibration Curve.

We generated 37 accelerator mass spectrometry radiocarbon dates and calibrated them using OxCal v4.3.2. Individuals are listed in order of mean calibrated radiocarbon date. Possible dates for each individual are indicated by the grey histogram (the higher the height of the histogram, the more likely that the individual dates to this time period). Source data are provided as a Source Data file.

Supplementary Note 1- Mitochondrial Haplogroup Determination via Multiplex PCR

We carried out mitochondrial DNA analysis of 76 skeletal samples from Roopkund at the Center for Cellular and Molecular Biology (CCMB) in Hyderabad, India using a multiplex PCR-based method.

Following the production of bone powder from each of the 76 bone samples (Table 2) using a sterile dentistry drill, we extracted DNA using a modified version of Yang, et al. [2]. We dissolved approximately 100mg of bone powder in 1.5 mL extraction buffer (0.5 M EDTA pH 8.0, 0.5% SDS, and 500 µg/mL proteinase K) and incubated in a shaking incubator at 37°C overnight. After spinning down each tube at 4000 rpm for 5 minutes, we discarded the pellet containing the cellular debris and transferred the supernatant containing the DNA to a 4mL Amicon filter (Sigma-Aldrich®). We brought the volumes down to 250ul by spinning the samples through the filters for 2-5 minutes. We transferred the supernatants to a 2mL Eppendorf tube containing 5X PB binding buffer (Qiagen®). We then transferred the samples to MinElute spin columns (Qiagen®), spun them at 7000 rpm for 1 minute, and discarded the eluate. We added 710 µL of PE wash buffer (Qiagen®) to the filter and spun the samples at 10,000rpm for 1 minute. After discarding the supernatant containing ethanol to wash off the salts, we dry-spun each tube at 14000 rpm for a minute to remove any trace ethanol. We discarded collection tubes and placed the filters in fresh 1.5mL Eppendorf tubes and added 45µL of EB elution buffer (Qiagen®) to the center of each filter. We followed this by incubation at 37°C for 15 minutes. After spinning the tube at 14000 rpm for 1 minute, we added an additional 30µL EB buffer to the same filter (to recover any leftover bound DNA molecules), incubated for another 10 minutes at 37°C and spun as above. We discarded the filter and retained the DNA eluate.

We amplified the mtDNA and genotyped it using the Sequenom iPLEX assay via the MassARRAY system (SEQUENOM, San Diego, CA), which requires a very small amount of input DNA (picogram scale) and is compatible with degraded, small-sized amplicons. Using this system, we

designed 4 panels (Supplementary Data 7) of amplification and extension primers, which target a total of 115 diagnostic mtDNA sites (23, 36, 31 and 25 sites, respectively). We performed multiplex PCR and genotyping according to the manufacturer's instructions. We report the genotyping results in Supplementary Data 8 for the 71 samples from which we were able to successfully extract ancient DNA. Assigned mitochondrial haplogroups are reported in Table 1 and Table 2.

Supplementary Note 2- Physical Anthropology Assessment of Roopkund Skeletons

The following section is an edited version of an unpublished report generated before genetic data were available by co-author Prof. Subhash Walimbe. The goal of our edits is to synthesize the anthropological discussions included in that report with the genetic findings. Newly added statements dealing directly with the genetic results are shown in italics. Some of the content of the original reports was used as part of the script of a National Geographic television documentary that was made describing the Roopkund Lake Site, so there are similarities between parts of the text that follows and that script[1].

The high-altitude (5029 meters) lake of Roopkund is situated in the eastern part of Chamoli District of Uttaranchal State. It nestles in the lap of Trisul (7,122 m), one of the highest peaks in India. The lake (*kund*) is also known as 'Skeleton Lake' for the puzzling occurrence of several hundred human skeletons in the lake itself and in the vicinity. The lake remains frozen for almost 11 months in a year, and when snow melts one can see these human skeletal remains, sometimes with flesh attached, well preserved in the alpine conditions.

At the time of the physical anthropology assessment and prior to the performance of genetic analysis, a set of five samples were analyzed by accelerator mass spectrometry (AMS) radiocarbon dating. Results are shown in Supplementary Table 1, and reveal that at least some of the samples at Roopkund Lake date to events approximately ~1200 years ago, *a result that we confirmed through radiocarbon dating performed in this study for the 23 individuals from the Roopkund_A cluster. An additional 14 individuals from the Roopkund_B and Roopkund_C clusters had much more recent dates of ~200 years ago (see main text). The fact that individuals from this temporal cluster happened not to be included in the first round of dating explains why the previous report does not discuss the presence of a later group of individuals.*

Supplementary Table 1. Radiocarbon dates obtained prior to the present study

OxCal ID	Sample ID*	Sample Type	13C (%)	Uncalibrated Date (years before 1950 CE)	95.4% calibrated interval
OxA-12792	Roopkund 11	tooth	-19.4	1145±50 BP	727-995 CE
OxA-12793	Roopkund 13	bone	-17.7	1241±27 BP	684-875 CE
OxA-12794	Roopkund 14	hair	-13.2	1200±26 BP	722-892 CE
OxA-12795	Roopkund 15	tooth	-13	1142±27 BP	777-978 CE
OxA-12796	Roopkund 16	tooth	-9.4	1240±28 BP	685-876 CE

Note: None of these sample IDs are ones for whom we obtained genetic results.

Many proposals have been suggested to explain the presence of skeletons at Roopkund Lake.

One proposal associates the skeletons to King Jasdhaval of Kannauj and his wife as well as their attendants. The theory is that the troupe perished in a blizzard following a Devi's wrath. This accident is suggested to have occurred around 1150 CE.

A second theory suggests that the skeletons are the remains of the Dogra General Zorawar Singh's army from Jammu which tried to invade Tibet in 1841, and which was beaten off and forced to find its way back home over the Himalayas.

Both these hypotheses are inconsistent with the five radiocarbon dates reported in Supplementary Table 1, which fall at neither of these times.

Anthropologists have known about the site since the early 1950s, and a few attempts were made to study these remains. Two expeditions travelled to Roopkund, one led by Prof. D. N. Mujumdar of Lucknow University, and the other by the Anthropological Survey of India, Kolkata. Both these teams estimated a population of 600-800 individuals who died at Roopkund Lake. The samples we studied anthropologically are randomly drawn from the skeletons.

Most individuals are represented in the anthropological analysis by complete neurocranial vaults (skull caps), or fragments thereof. Although preservation of the bones is excellent (there is no weathering), no skull is complete. No significant bones of the facial region were found in articulation with the neurocranial vault (except in one or two cases, where nasal bones are *in situ*). There were around 20 complete or fragmentary gnathic bones, maxilla or mandible, with some teeth preserved in their sockets.

A set of 25 complete long bones, 3 broken long bones, and 3 girdle bones were also available for study. No bone of the thoracic cage, except 12 vertebrae and one sacrum, were analyzed.

Prof. Bhattacharya of Delhi University studied the phenotypic features of the skeletons. In his opinion articulated at the time of the original composition of this anthropological report (and thus prior to the availability of genetic data), there are two distinct groups of individuals: one with very robust features, and the other with moderate features. For the 'robust' group, the robusticity is evident in the form of prominently developed features on the cranium, such as a prominent glabellar region (the central region immediately above the orbits), supra-orbital ridges, prominent temporal lines, occipital crest (where neck muscles are attached), and large mastoids. These are all places that provide surfaces for muscle attachment. The long bones, especially the femora from the Lucknow collection and humeri from the Pune collection, are extremely robust. On the other hand, there is also a 'not so robust' group with less well-developed features.

Twenty-five complete long bones permit estimation of stature. Four individuals are very tall, with height in the range 184.41-187.55 cm. It is likely that these are males. Except for three individuals with statures of 178.16 cm, 181.33 cm, and 179.79 cm, respectively, all other individuals are below 175 cm in height. The minimum height recorded for the series is 145 cm. Some of the 'short' individuals could be females. Inference of sex is based on robusticity, and because males tend to be more robust and females, there is some uncertainty in this classification. Nevertheless, there are at least two cases (in the Lucknow collection) where

confirmation of sex is based on the morphology of a wide sciatic notch, which unmistakably indicates female sex. Evidence of several shell bangles both in the Lucknow and Pune collection can be taken as supplementary pieces of evidence confirming that the Roopkund population consisted of both males and females. *After the compilation of this physical anthropology report, we confirmed the presence of many females among the Roopkund individuals, with 23 genetic males and 15 genetic females (see the main text).*

Does the differential expression in robusticity fall within the normal range of variation in a homogeneous population? This question is difficult one to answer. Is either of the sets of skeletons--the robust or the delicate ones--consistent with being local? Prof. Bhattacharya's study on morphological assessment of the series is important in this regard, and the following information may shed some light on these questions.

One of the major works undertaken by the Anthropological Survey of India is the study of biological variation in Indian populations. Most Indian communities have a stature of around 163-165 cm. There are few communities reported to have a typical stature of more than 170 cm, and they include Gujjar Muslims of Saho-Chamba (Himachal Pradesh) at 174 cm, Burishki-Hunza of Jammu-Kashmir at 171 cm, Maharatta Coorg of Karnataka at 174 cm, Moplas Sunni Muslims of Kerala at 176 cm, Jats and Sikhs of Punjab at 172-180 cm, Jats of Meerat at 174-182 cm, and Rajput (Dogra-Hindu-Muslim-Marwari groups) of the Indo-Gangetic Plain at 175-182 cm. No Indian population, except these, is known to have such a large average height.

In this regard the Roopkund group is tall: except for individuals 18, 19, 24 and 25 (of which 18 and 25 are positively females), all have a 'more than average' stature. It must, however, be remembered that the above are averages and in every population there are exceptionally tall and strong individuals. Going to such a high altitude would require a minimum level of toughness and therefore individuals having stature up to perhaps 170-172 cm (5'6"-5'7") might be unsurprising (for example, they could be a local group of porters). The 'sturdy' group is of individuals having stature of more than 175 cm, which were almost certainly 'visitors' as their

height is very atypical of peoples of the region. *It is of course tentative to hypothesize anything on the basis of robusticity alone. Nevertheless, it is notable that the physical anthropologists who compiled the original version of this report emphasized—prior to the availability of genetic data—that the variation in robustness of some of the individuals raised the possibility that the individuals coming from multiple populations, consistent with the later genetic findings. Although we do not have long bone length measurements on the individuals on whom we obtained DNA, it is tempting to hypothesize that the tall and robust individuals correspond to the Roopkund_B cluster of Mediterranean origin.*

The Roopkund series is predominantly of middle-aged adults (35 to 40 years). There are young adults (around 18-20 years of age) too. One skeleton of an individual of around 15 to 16 years of age was noticed at the site. In this case, epiphyseal fusion for distal ends of leg bones, femur and tibia, had occurred just prior to the death. There are no immature babies or children so far noted from the site. There are a few very old individuals in the series as well (definitely 50+ years). Cranial sutures in these individuals are completely fused and the teeth show significant attrition even on the third molars. The presence of elderly ladies in the series is noteworthy. It is consistent with the notion of pilgrims and inconsistent with the 'army' hypothesis.

The Roopkund individuals overall appear very healthy. Even in old individuals, there is no significant noticeable pathology. There are few vertebrae in the collection, so no comment can be made on possible 'degenerative pathologies' like spondylitis or vertebral lipping. Long bone ends do not show any indication of arthritic lesions. Even osteoporosis, the most common skeletal metabolic disease in older individuals, is not reflected in any of the bones. This disease is primarily characterized by a reduction in total bone volume caused by thinning of cortical walls of the long bones; the Roopkund long bones are extremely strong.

The oral health of the Roopkund series is good. Except for the natural wear caused by masticatory stress there is no other pathology. The only exception was an alveolar abscess in one specimen above the maxillary first molar (which is lost, probably because of caries infection

long before the individual died). There are few cases of antemortem tooth loss in the old aged specimens.

There are some indications of nutritional stress, signs of which are seen on bones in the form of porosity and porotic hyperostosis. There are two possible deficiencies consistent with the data.

The first explanation is iron deficiency anaemia, that is, reduction in concentration of haemoglobin and/or red blood cell counts below normal. Iron is needed for the development of haemoglobin in newly formed RBCs (produced in bone marrow), so in anaemia RBCs become pale and small and have a much shorter life span. This can contribute to abnormalities in the skeleton. The main diagnostic criteria are thinning of the outer table of the skull and thickening of the diploe between two skull tables (reflecting the body's attempt to produce more RBC by increasing the marrow space). When orbital margins are affected, the lesion is called as cribra orbitalia. The changes on the orbital roof in the form of 'holes' seen on some specimens can be misunderstood as cribra orbitalia (caused by iron deficiency). However, since there are virtually no changes in the cranial bones (such as thickening) the diagnosis of iron deficiency (as stated in the earlier reports) is unlikely to fully explain these observations. The porosity on frontal and parietal bones seen in many other specimens could, however, be anaemic in origin.

Another possible explanation for the porosity in these bones might be a vitamin deficiency, for example a deficiency in Vitamin C which is necessary for the body to combat infection, for normal formation of body tissue, and for the absorption of iron. In addition to reducing the resistance to infection, vitamin C deficiency predisposes to bleeding into the skin and beneath the periosteum (membrane surrounding the bones). The result is the formation of new bone on the skeleton as a response to bleeding. Jaw bones are generally the most affected elements, as the gums tend to bleed while chewing food. We have only a few maxillary and mandibular fragments. Yet on one maxillary sinus floor there is an evidence of new bone formation (which could be maxillary sinusitis, as well). The changes in the orbital region, characteristically new bone formation (in more understandable terms 'scurvy'), are positive indications of vitamin C

deficiency. A prolonged winter and inhospitable climate around Roopkund Lake and the consequent absence of fresh produce for many months would contribute to such changes.

There are also many cases of 'warping' in the Roopkund series. Depression near the bregma seen in one case or depression in the post-bregmatic region in the other case could be a result of low weight pressure for extended periods of time. Individuals who spent large amounts of time carrying loads on the head could be expected to have such skeletal patterns.

There are examples of cracks on many crania that probably occurred years after death. These cuts are sharp, with slight chipping of bone along the edges and running for up to 8 to 10 cm in length along the parietal or frontal bones. Such damage is not expected to occur when there is soft tissue cover. A possibility is that they reflect landslides causing rolling of defleshed skeletons.

There are three cases that require special mention: a depression near the lambdoid region in one individual affecting an approximately 5x5 cm area, a depression on the orbital roof in another individual, and an ‘injury’ on the frontal bone of the third individual. These can be described as ‘compression fractures’ which are caused by excessive impacts. The very fact that some broken fragments are still attached to the skull and unhealed indicates that the incidents occurred at or near the time of death (or could even be the cause of death). A blow by a heavy object like a stone of the size of a cricket ball can cause such an injury.

Can Roopkund’s weather and topography provide a clue to explain these compression fractures? The area around Roopkund is known to be regularly hit by hailstones as large as cricket balls. It is also interesting to note that in oral traditions, the local folk women sing of an ancient tale, of how the Hindu Goddess Nanda Devi, angered by her devotees, rained down a storm of hail as hard as iron, killing them all on the spot. This would be consistent with at least some of the Roopkund individuals dying as a large group of people, both men and women, was winding its way past Roopkund Lake, and encountered a hailstorm from which it could not take

shelter, resulting in the death of large parts of the party. *In light of the genetic data and new radiocarbon dates, a single deadly event could not be the whole story of what happened at Roopkund Lake, although it remains a reasonable suggestion that hailstorms explain some of the deaths, epsecially in light of the three individuals with compression fractures.*

Supplementary Note 3- Genetic screening for ancient pathogens

One possible cause of death of the individuals at Roopkund Lake is that they were the victims of an epidemic. To search for evidence of this, we screened the genetic data for signs of bacterial pathogens which may indicate cause of death[3].

While our procedure for generating ancient DNA data was specifically designed for enriching human DNA, there is still a large proportion of non-human sequences associated with each sample; in our data, an average of 74% of sequences from the libraries made from Roopkund samples do not map to any of the 1.2 million SNP sites[4-7] that were targeted during the human enrichment capture (range: 46-93%) (Supplementary Table 2). While much of this DNA can be assumed to be the result of environmental contamination, it is also possible to identify DNA from ancient bacteria that were present in the individual at the time they died.

We used MALT[3] to screen all sequences (after merging and removal of duplicates) for evidence of bacterial pathogens in each individual. MALT compares each sequence to the entire NCBI bacterial database, using an algorithm to specifically assign each sequence to a particular node representing a species or group of species in the bacterial species tree[3]. We then performed a series of validation steps using the tool HOPS[8] to assess the authenticity of sequences assigned to 165 nodes of interest (Supplementary Table 2), selected based on their relationship to common human pathogens. The sequences are assessed for authenticity using several criteria[9], including:

1) **Edit distance**: A measure of the number of differences in nucleotide identity between the sequence and the reference sequence to which it is aligned. When a pathogen is present in a sample, we would expect that the majority of aligned sequences to be extremely similar to the reference sequence (low edit distance). We therefore require

that the distribution of edit distances be approximately exponentially decreasing for a particular pathogen node to be considered plausibly authentic.

2) **Ancient DNA damage**: Authentic ancient DNA has characteristic cytosine deamination especially at the terminal ends of molecules. We therefore filter out all sequences that do not contain a C-T (cytosine-thymine) mismatch relative to the reference genome.
3) **Edit distance of damaged sequences**: We examine the edit distance distribution of all sequences that pass the ancient DNA damage filter at each node. Again, we expect that authentic, pathogenic ancient DNA would be similar to the reference sequence (after accounting for the minimum of 1 mismatch due to the C-T mismatch used to ascertain these sequences). We require that the distribution of edit distances is exponentially decreasing (starting at edit distance = 1, rather than 0) in order to consider the node to be plausibly authentic.

The confidence with which we can detect bacterial DNA for each sample is scored based on the number of authentication stages that it passes (e.g. if a sample that has an exponentially distributed edit distance, but no ancient DNA damage, it will be classified as stage 1) using the tool HOPS[8]. Samples that have aligned sequences to any of the pathogen nodes of interest that pass at least stage 1 are included in Supplementary Figure 2.

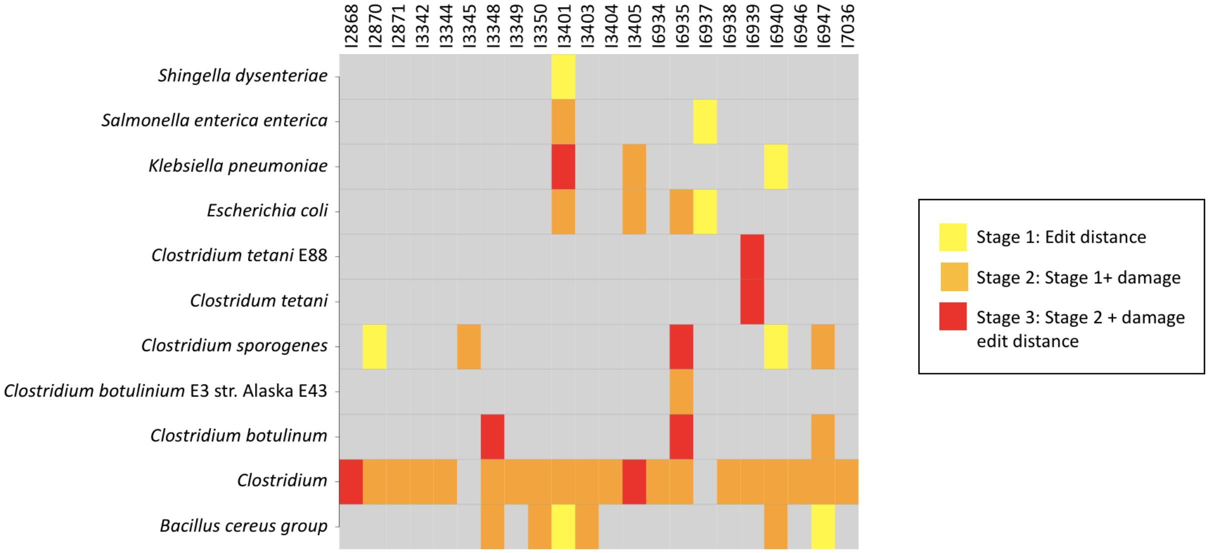

Supplementary Figure 2 Heatmap of positive pathogen screening hits. This heatmap shows all positive hits for pathogens at nodes of interest that minimally meet the stage 1 screening criteria. The highest authentication stage passed is indicated by the color (stage 1: yellow, stage 2: orange, stage 3: red), with nodes that did not meet authentication stage 1 shown in grey.

We manually assessed all positive hits (of stage 1 or greater) using criteria such as number of aligned sequences, distribution across the pathogen genome, sequence length, and duplication rate, in addition to edit distance and damage profile to determine whether there is any evidence for authentic ancient pathogen DNA.

While we detect a number of plausible occurrences of the Clostridium bacteria in the data, we find no evidence of bacterial pathogens that may provide an explanation for the cause of death of the individuals at Roopkund. Clostridium is a common soil bacterium[10], and its presence among numerous samples and the wide variety of different Clostridium nodes with positive hits suggests that this bacterium is likely present in the environment at Roopkund. No other pathogens appear to show convincing evidence of presence in the ancient DNA samples. We note that while we have been unable to detect human pathogens in the Roopkund data, we cannot exclude the possibility that other undetected pathogens may have been responsible for the death of the Roopkund individuals.

Supplementary Table 2 Bacterial pathogens potentially detected in the screen

African swine fever virus	Herpes simplex virus (type 1 / strain F)	Salmonella enterica subsp. enterica
Aspergillus fumigatus	Herpes simplex virus (type 1 / strain HFEM)	Salmonella typhimurium TR7095
Bacillus anthracis	Herpes simplex virus (type 1 / strain HZT)	Schistosoma mansoni
Bacillus cereus group	Herpes simplex virus (type 1 / strain MGH-10)	Shigella boydii
Bartonella bacilliformis	Herpes simplex virus (type 1 / strain MP)	Shigella dysenteriae
Bartonella henselae	Herpes simplex virus (type 1 / strain Patton)	Shigella flexneri
Bartonella quintana	Herpes simplex virus (type 1 / strain R19)	Shigella sonnei
Blastomyces dermatitidis	Herpes simplex virus (type 1 /strain RH2)	Staphylococcus aureus
Bordetella	Herpes simplex virus (type 1 / strain SC16)	Streptococcus
Bordetella pertussis	Herpes simplex virus unknown type	Streptococcus gordonii
Bordetella petrii	Histoplasma capsulatum	Streptococcus gordonii str. Challis substr. CH1
Borreliaceae	Klebsiella oxytoca	Streptococcus mutans
Borreliella afzelii	Klebsiella pneumoniae	Streptococcus pneumoniae
Borreliella burgdorferi	Klebsiella pneumoniae subsp. rhinoscleromatis	Streptococcus pyogenes
Borreliella garinii	Legionella pneumophila	Taenia solium
Brucella	Leptospira alexanderi	Tannerella forsythia
Brucella abortus	Leptospira alstonii	Tannerella forsythia 92A2
Brucella melitensis	Leptospira borgpetersenii	Toxoplasma gondii
Brucella microti	Leptospira broomii	Treponema
Brucella ovis	Leptospira fainei	Treponema denticola
Brucella suis	Leptospira inadai	Treponema denticola ATCC 35405
Burkholderia cepacia	Leptospira interrogans	Treponema pallidum
Burkholderia mallei	Leptospira kirschneri	Treponema pallidum subsp. pallidum
Burkholderia pseudomallei	Leptospira kmetyi	Treponema pallidum subsp. pallidum
Chlamydia abortus	Leptospira licerasiae	Treponema pallidum subsp. pertenue
Chlamydia caviae	Leptospira noguchii	Trichinella spiralis
Chlamydia felis	Leptospira santarosai	Trypanosoma brucei
Chlamydia muridarum	Leptospira weilii	Trypanosoma cruzi
Chlamydia pecorum	Leptospira wolffii	Variola major virus
Chlamydia pneumoniae	Methanobrevibacter oralis	Veillonella parvula
Chlamydia psittaci	Moraxella catarrhalis	Vibrio cholerae
Chlamydia trachomatis	Mycobacterium abscessus	Vibrio parahaemolyticus
Clostridium	Mycobacterium africanum	Vibrio vulnificus
Clostridium botulinum	Mycobacterium avium	Yersinia enterocolitica
Clostridium botulinum BKT015925	Mycobacterium bovis	Yersinia pestis
Clostridium botulinum E3 str. Alaska E43	Mycobacterium canettii	Yersinia pseudotuberculosis
Clostridium sporogenes	Mycobacterium colombiense	Yersinia pseudotuberculosis complex
Clostridium tetani	Mycobacterium indicus pranii	
Clostridium tetani E88	Mycobacterium intracellulare	
Coccidioides immitis	Mycobacterium kansasii	
Coccidioides posadasii	Mycobacterium leprae	
Corynebacterium diphtheriae	Mycobacterium tuberculosis complex	
Coxiella burnetii	Mycoplasma pneumoniae	
Cryptococcus gattii	Neisseria	
Cryptococcus neoformans	Neisseria gonorrhoeae	
Cynomolgus Epstein-Barr Virus A4	Neisseria meningitidis	
Cynomolgus Epstein-Barr Virus Si-IIA	Parvimonas micra	
Cynomolgus Epstein-Barr Virus TsB-B6	Peptostreptococcus anaerobius	
Enterobius vermicularis	Peptostreptococcus micros CC57A	
Epstein-barr virus strain ag876	Plasmodium falciparum	
Epstein-barr virus strain p3hr-1	Plasmodium vivax	
Escherichia coli	Pneumocystis carinii	
Escherichia coli O111	Pneumocystis jirovecii	
Escherichia coli O157:H7	Porphyromonas gingivalis	
Escherichia coli O26	Porphyromonas gingivalis W83	
Haemophilus influenzae	Rickettsia africae	
Haemophilus influenzae Rd KW20	Rickettsia akari	
Helicobacter pylori	Rickettsia conorii	
Herpes simplex virus 1 strain R-15	Rickettsia felis	
Herpes simplex virus (type 1 / strain 17)	Rickettsia japonica	
Herpes simplex virus (type 1 / strain A44)	Rickettsia prowazekii	
Herpes simplex virus (type 1 / strain Angelotti)	Rickettsia rickettsii	
Herpes simplex virus (type 1 / strain CL101)	Rickettsia sibirica	
Herpes simplex virus (type 1 / strain CVG-2)	Rickettsia typhi	

Supplementary Note 4- Stable carbon and nitrogen isotope analysis of bone collagen

The analysis of stable carbon ($\delta^{13}C$) and nitrogen ($\delta^{15}N$) isotope ratios of bone collagen is regularly utilized as a method of palaeodietary reconstruction in archaeology [11-15]. The method is based on the fact that stable carbon and nitrogen isotope ratios in bone collagen reflect the stable carbon and nitrogen ratios of the diet, and that different food sources can be differentiated into groups on the basis of these ratios[12,16,17]. Controlled diet experiments with laboratory rats have shown that owing to preferential routing, bone collagen $\delta^{13}C$ and $\delta^{15}N$ values primarily reflect dietary protein and not the whole diet [18].

Stable carbon isotope analysis primarily focuses on the variability of carbon isotope ratios between different groups of primary producers in the environment which is linked to isotopic fractionation during photosynthesis. Since the lighter isotope, ^{12}C, diffuses faster than the heavier ^{13}C, all plants are enriched in ^{12}C compared to ^{13}C, albeit to different degrees. Three different photosynthetic pathways allow the grouping of plants on the basis of this enrichment. The C_3 or Calvin-Benson pathway is found in most plant species, including crops such as wheat, rice, barley and potato. In C_3 plants the $\delta^{13}C$ varies between -24 to -36‰ [19,20]. The C_4 or Hatch-Slack pathway is commonly seen in arid-adapted plants, and crops such as maize, sorghum and millet. The $\delta^{13}C$ for such plants averages -12.5‰[19,21]. A small proportion of plants utilize the Crassulacean Acid Metabolism (CAM) pathway and these demonstrate tremendous variability in isotopic fractionation[19]. In marine ecosystems, the primary producers demonstrate relatively enriched ^{13}C, which affects the whole trophic chain. As a result, consumption of marine food sources results in elevated $\delta^{13}C$ values compared to terrestrial-based diets [12].

Animals (including humans) consuming these plants reflect these isotopic differences, so that stable carbon isotope analysis of their tissues allows inferences about the diet of past individuals[18]. Stable nitrogen isotope analysis of these tissues provides further dietary

resolution. $\delta^{15}N$ values indicate the trophic level of an organism within its ecosystem, with an average of 3-5‰ trophic enrichment in ^{15}N from food source to consumer[17,22]. However, $\delta^{15}N$ values within an ecosystem are also affected by factors such as manuring of crops, aridity, physiology, soil salinity, and weaning[23-25]. In marine ecosystems, the trophic level enrichment results in high $\delta^{15}N$, distinguishing them from terrestrial-based diets [26].

Stable isotope measurements for all 45 bones analyzed in this study, including 37 samples for which we also report ancient DNA, are reported in Supplementary Data 4.

Supplementary Note 5- Relationship of Roopkund_B to individuals from present-day Crete

The analyses reported in this study highlight a close relationship between the Roopkund_B group and individuals from present-day Crete (Crete.DG). A *qpWave* analysis of Roopkund_B and 26 present-day groups from around the world indicates that Roopkund_B is consistent with being a genetic clade with individuals from Crete with respect to all other groups included in the analysis. However, as this analysis was only performed a subset of the groups for which we have genetic data, we sought to test this in a more rigorous way.

We first attempted to determine whether Roopkund_B shows greater affinity for the population from Crete than to any other groups in the dataset, using the statistic f_4(Roopkund_B, South_Africa_2000BP; Crete.DG*, Test*), where *Test* is all other populations in the dataset. This statistic is expected to be significantly negative in cases where Roopkund_B shows more affinity to the *Test* population than to Crete.DG. We observe multiple cases where the statistic is significantly negative (more than 3 standard errors away from zero) (Supplementary Figure 3a; Supplementary Data 9), particularly when *Test* is an ancient population from present-day Eastern Europe.

To obtain insights into the nature of the differences in ancestry observed between Roopkund_B and individuals from Crete, we use the statistic f_4(South_Africa_2000BP, *Test*; Crete.DG, Roopkund_B). This statistic tests whether Crete.DG and Roopkund_B behave as a true genetic clade with respect to all other populations in the dataset. In cases where the *Test* population is more closely related to Roopkund_B than Crete.DG, the statistic will appear significantly positive, while in cases where the reverse is true, the statistic will be significantly negative. We observe a number of instances where the statistic is significantly different from 0 (Supplementary Figure 3b; Supplementary Data 10). The statistic is most significantly positive in cases where *Test* represents a population of ancient Eastern European or Near Eastern ancestry.

Finally, we use f_3-statistics to determine whether modeling the ancestry of Roopkund_B using the Crete.DG population may require admixture from an additional source population. We computed statistics of the form f_3(Roopkund_B; Crete.DG, *Test*), which can be significantly negative only if Roopkund_B is admixed with ancestry related (perhaps distantly) to both Crete.DG and the *Test* population. We observe 4 instances in which the statistic is significantly negative (Supplementary Figure 3c; Supplementary Data 11), the most significant of which involve a *Test* population that is ancient Eastern European or Near Eastern in origin, providing further evidence that the Roopkund_B population is admixed.

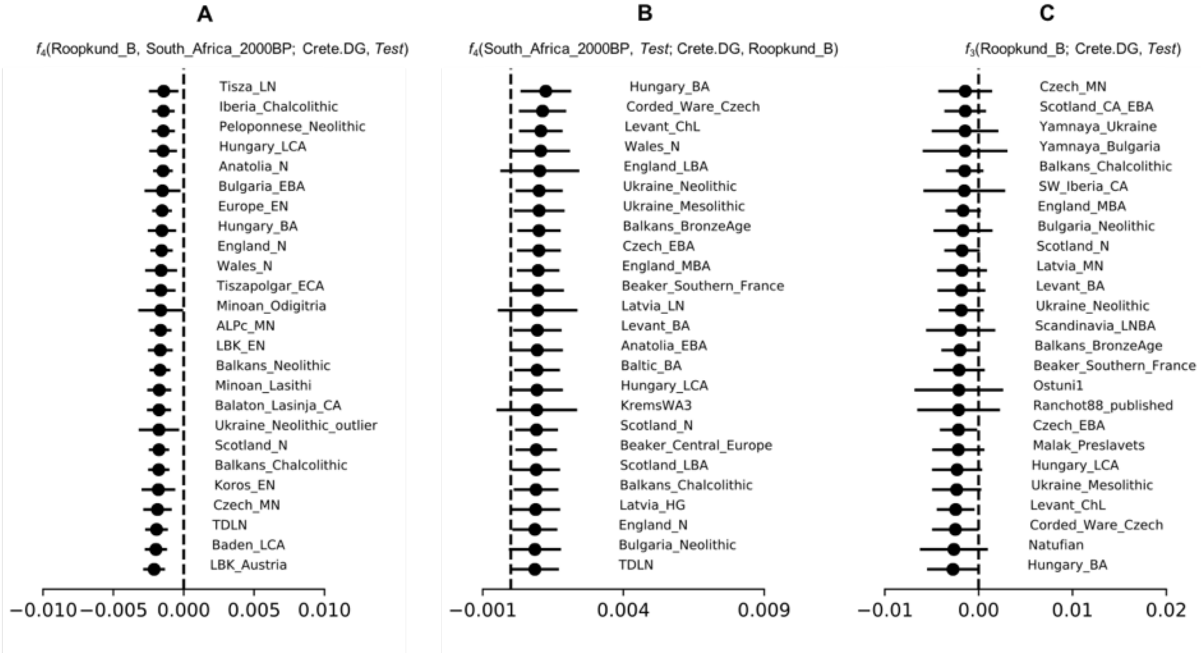

Supplementary Figure 3 *f*-statistics (A) The statistic f_4(Roopkund_B, South_Africa_2000BP; Crete.DG, *Test*) measures the relationship between Roopkund_B and *Pop*. The statistic is most negative in cases where Roopkund_B shares a greater affinity for *Test* than *Pop*. (B) The statistic f_4(South_Africa_2000BP, *Test*; Crete.DG, Roopkund_B) tests whether Roopkund_B and *Pop* behave as a clade. The statistic is most positive in cases where Roopkund_B shares a greater affinity for *Test* than *Pop*. (C) The statistic f_3(Roopkund_B; Crete.DG, *Test*) is significantly negative in cases where the target population, Roopkund_B, is admixed, and requires ancestry from both Pop and Test populations in order to model its ancestry. In each panel, the 25 *Test* populations that produce the most significantly negative or positive statistics are shown. Error bars represent ±3 standard errors. Source data are provided as a Source Data file.

These analyses indicate that while Roopkund_B is closely related to the population from present-day Crete, the two groups do not belong to a homogenous genetic clade. Instead, Roopkund_B possesses additional ancestry that is slightly more related to other populations, particularly those from ancient Eastern Europe or the Near East. The population from Crete represents the best available proxy for the ancestry observed in the Roopkund_B group, but analysis of additional data from ancient populations that are contemporaneous with Roopkund_B may reveal a better fitting source of the ancestry of these individuals.

Supplementary Note 6- Modeling the ancestry of Roopkund_A and Roopkund_C individuals

While the homogenous ancestry observed in the Roopkund_B group makes it possible to model the group's relationship with other present-day populations using *qpWave*, the heterogeneous composition of the Roopkund_A group makes such an analysis less well defined. We applied the *qpWave/qpAdm* methodology [6,27,28] to model the ancestry of each individual in the Roopkund_A group separately (as well as the Roopkund_C individual).

We first attempt to determine whether each Roopkund_A individual is consistent with being a genetic clade relative to any present-day population using *qpWave*.[27,28] In this case, the present-day population comparison dataset includes 14 present-day populations (Brahmin_Tiwari, Chukchi, French, Han, Karitiana, Mala, Mbuti, Onge, Papuan, Bengali, Palliyar, Irula, Baniyas, and Kalash). We find that some individuals can be plausibly modeled as a genetic clade with one or more of these populations (Supplementary Figure 4; Supplementary Data 12). As it was not possible to plausibly model all Roopkund_A individuals using this method, we also applied the related *qpAdm* methodology [6] to attempt to model each individual as related to any two of the selected populations via admixture (Supplementary Data 13). We also computed a published f_4-ratio statistic[27] of the form f_4(Yoruba,Basque;*Test*,Onge)/f_4(Yoruba,Basque;Georgian,Onge) to infer the proportion of West Eurasian ancestry in the Roopkund_A individuals as well as in present-day Indian populations, and found that groups that formed clades with the individual ancient samples tended to have similar West Eurasian-related ancestry proportions (Supplementary Data 14).

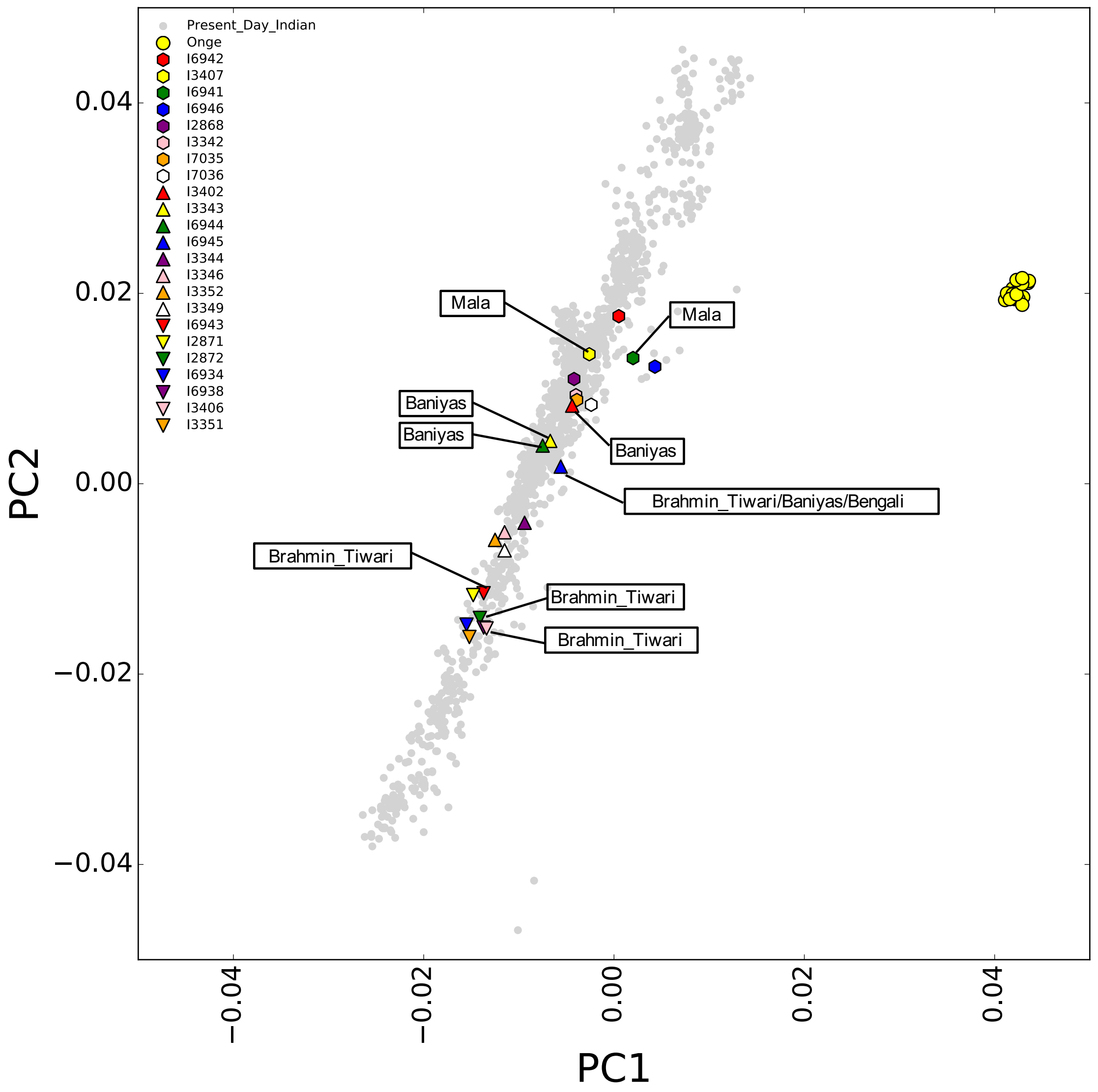

Supplementary Figure 4 Indian Cline PCA. A zoomed in version of the Indian Cline PCA (Figure 2a in the main text), with Roopkund_A individuals assigned different markers. Individuals that could be modeled as a genetic clade with one or more populations in the *qpWave* analysis are labeled with the population with which they could be modeled.

We also apply the same method to the Roopkund_C individual, this time use the comparison set Brahmin_Tiwari, Chukchi, French, Han, Karitiana, Mala, Mbuti, Onge, Papuan, Bengali, Brahmin_Nepal, Japanese, Korean, Malay, Tibetan.DG, and Vietnamese. We are unable to model the Roopkund_C individual as a genetic clade with any of these populations

(Supplementary Data 15), and therefore perform a *qpAdm* analysis to determine whether it is possible to model Roopkund_C as related to any two of these populations via 2-way admixture. The only such model that is plausible assigns approximately 82% of the ancestry of the Roopkund_C individual to a population related to present-day Vietnamese, and the remaining 18% to a population related to present-day Malay (Supplementary Data 16). These results suggest that Roopkund_C individual is likely of Southeast Asian origin.

We also repeated these analyses adding in the population Cambodian.DG to the outgroup set. Doing so results in the rejection of the model that Roopkund_C is a mixture of groups related to Malay and Vietnamese (Supplementary Data 17). This suggests that the Roopkund_C individual shares some genetic drift with the Cambodian.DG population that is not shared with either Malay or Vietnamese. However, a *qpAdm* model with Cambodian.DG and Malay as sources has a passing p-value (0.18) albeit with implausible admixture proportions of 123% from Cambodian.DG and -23% from Malay. A possible interpretation of this is that the Roopkund_C individual falls on a genetic cline that includes the Cambodian.DG and Malay populations, but that Roopkund_C falls in a more extreme position on the cline, beyond the position of the Cambodian.DG individual. There is known South Asian-related admixture in Cambodian populations and interaction between Cambodia and South Asia is also evident in the material culture record as reflected for example in the South Asian-influenced culture that built Angkor Wat[29]. Putting the genetic and cultural information together, we hypothesize that the Roopkund_C individual of Southeast Asian-related ancestry could have been from a group that was in cultural and genetic contact with South Asia like Cambodians, but that it has additional South Asian-related ancestry beyond what is observed in any of the Southeast Asian populations included in the present analysis. This evidence that the Roopkund_C individual was probably from a southeast Asian group in cultural contact with South Asia adds richness to our observation that the individual died near an important Hindu shrine at around ~5,000 meters in the high Himalayas.

Supplementary Note 7 - No affinity of Roopkund_A to modern Himalayan groups

In order to determine whether the skeletons of Roopkund are genetically related to present-day populations that neighbor the Indian Himalayas, we generated genome-wide data from 5 populations, using two different platforms. We obtained blood samples with informed consent of donors following practices reviewed by the Institutional Review Board of the Centre for Cellular and Molecular Biology in Hyderabad India. We obtained genome-wide SNP genotyping data from 88 individuals from the highlands of the Leh and Ladakh regions of Jammu and Kashmir using the Illumina Global Screening Array. These samples will be referred to collectively using the population label 'Ladakh'. We genotyped an additional 16 samples from 4 groups from four villages that are nearby to Roopkund Lake (Dewal: n=5, Baimaru: n=5, Wan: n=3, Tiirpak: n=3) using the Affymetrix Genome-Wide Human SNP Array (6.0).

The Illumina Global Screening Array generated genome-wide information from 635,700 sites, while the Affymetrix Genome-Wide Human SNP Array yielded genome-wide data from 909,622 sites. To maximize the number of sites analyzed, we treated these two datasets separately. We separately merged each dataset the 591,304 SNP dataset described in the main text using the program mergeit [30] with default parameters, including docheck: YES and strandcheck:YES. To ensure that different SNP naming strategies between the sequencing platforms did not result in data loss during merging, we considered SNPs to be the same if they had identical genetic positions, except in cases where the described alleles did not match, or where it was not possible to determine the strand of each allele (i.e. C/G and A/T). This resulted in two merged datasets with 54,565 SNPs in the case of the Illumina Global Screening Array and 162,341 SNPs in the case of the Affymetrix Genome-Wide Human SNP Array.

To determine the relationship between these newly sequenced samples and the Roopkund samples, we regenerated the "Indian Cline" Principal Component Analysis (PCA) plot described

in the main text for each dataset, using *smartpca* [30] with default parameters, in addition to the settings lsqproject:YES and numoutlier:0. The PCA included 1,453 present-day populations defined in Nakatsuka, et al. [31]. We projected the Roopkund individual and the newly reported present-day Himalayan individuals.

We find that the individuals from Ladakh do not cluster along the "Indian Cline" (Supplementary Figure 5a). Instead they form a broad cline along PC1, suggesting that some individuals have more East Asian-related ancestry than is observed in the other present-day Indian groups included in our analysis. Although some individuals from this sample fall close to some of the Roopkund_A and Roopkund_C individuals, the ancestry of the Roopkund samples is not well described by the heterogeneous ancestry of the Ladakh individuals.

We observe a similar excess of East Asian-related ancestry in the individuals from villages that neighbor Roopkund Lake (Supplementary Figure 5b). These individuals do not appear to have ancestry related to any of the groups from Roopkund, and do not form a tight cluster, and their positioning on the plot does not appear to be correlated with their village, suggesting that the ancestry of individuals from around Roopkund Lake is relatively heterogeneous.

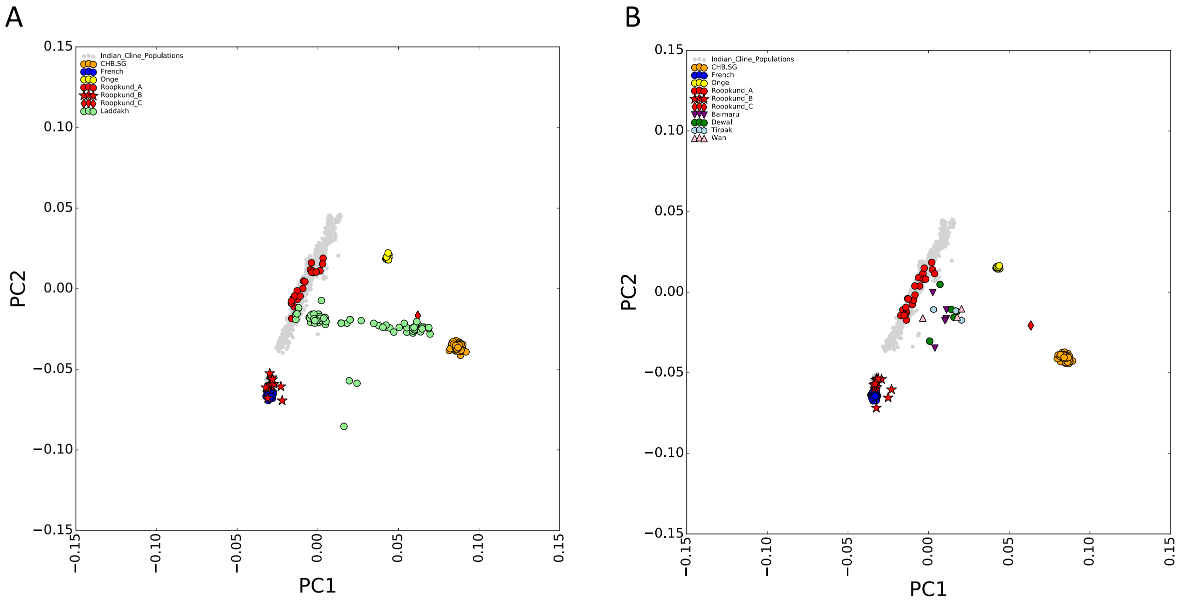

Supplementary Figure 5 Principal component analysis of 1,453 present day individuals from populations throughout India (highlighted in grey), in addition to French (highlighted in blue), Han Chinese from Beijing (CHB; highlighted in orange), and Onge (highlighted in yellow). In each plot, the 38 Roopkund individuals are projected onto the first two principal components. The individuals from Roopkund are grouped into three distinct categories (Roopkund_A, Roopkund_B, and Roopkund_C) based on their position in the PCA. (A) Present-day individuals from Ladakh are projected. (B) Present-day individuals from Baimaru, Dewal, Tiipak, and Wan are projected. Source data are provided as a Source Data file.

We also computed pairwise F_{ST} between the newly described populations and all other populations in the dataset (Supplementary Data 18; Supplementary Figure 6). We find that the individuals from the four sampled villages (Dewal, Tiirpak, Wan, and Baimaru) exhibit little population differentiation (average pairwise F_{ST}=0.007). The people of these villages do not appear to exhibit a particular affinity to either of the Roopkund groups, showing similar levels of affinity to Roopkund_A as they do to other present-day Indian populations (Brahmin_Tiwari and Mala) and to Roopkund_B as they do to Crete.DG. A similar pattern is apparent for the Ladakh samples.

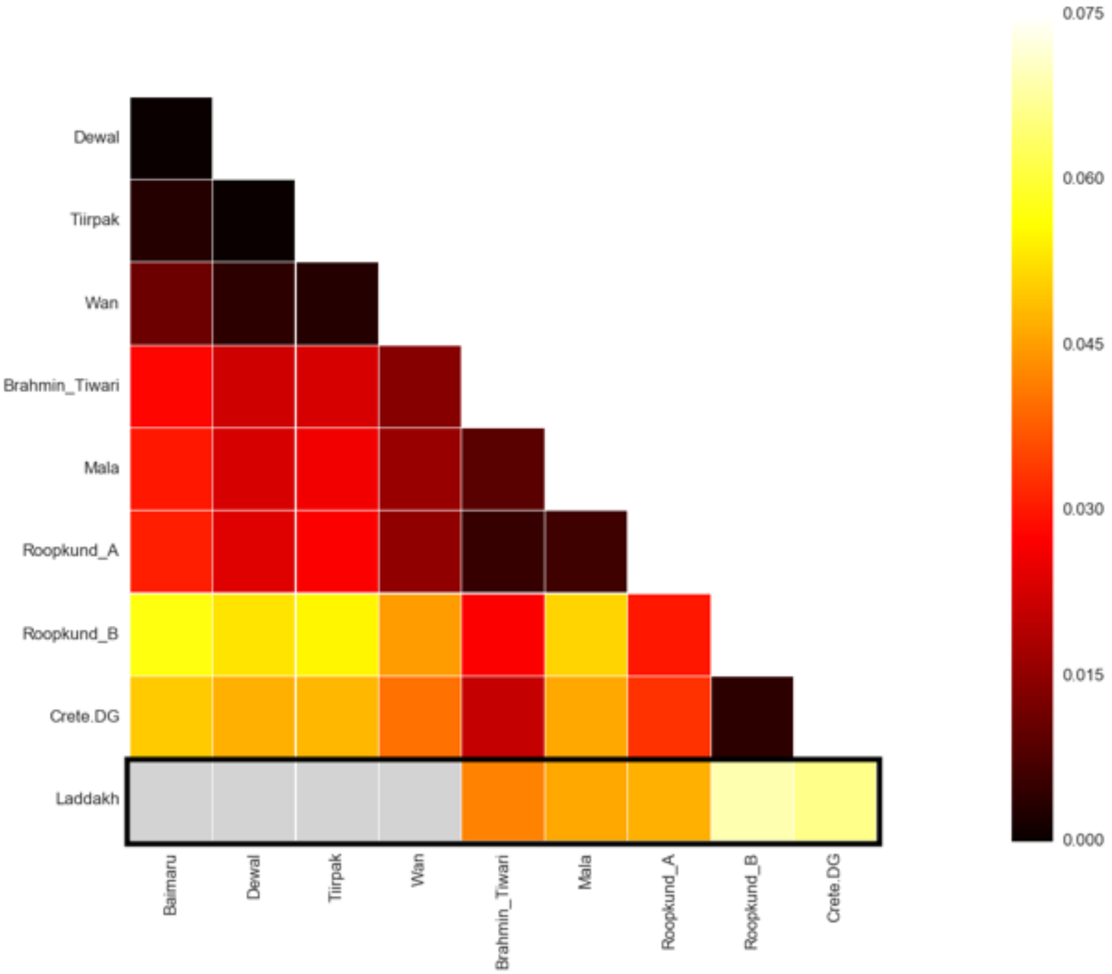

Supplementary Figure 6. Pairwise F_{ST} between the newly reported present-day Indian groups (Baimuru, Dewal, Tiirpak, Wan, and Ladakh) and Brahmin_Tiwari, Mala, Roopkund_A, Roopkund_B, and Crete.DG. All comparisons with the Ladakh population (enclosed in a black border) are made using the merged Illumina Global Screening Array dataset, while all other comparisons made using the merged Affymetrix Genome-Wide Human SNP Array dataset. Darker colors indicate greater affinity between comparison populations. Missing comparisons are shown in grey. Source data are provided as a Source Data file.

Supplementary Note 8- Systematic ancestry differences in Roopkund_A males and females

We were struck by the large proportion of genetically female individuals identified in the dataset and were curious whether it was possible to detect any systematic differences in ancestry between males and females each of the two Roopkund groups, as this may provide further clues about the identity and purpose of these travelers.

We visually examined the relative placement of genetic males and females from each group on the same PCA plots presented in Figure 2 of the main text, distinguishing between the two sexes using marker color. We first examine the placement of the Roopkund_A individuals on the "Indian Cline" PCA (Supplementary Figure 7a). While male and female individuals are scattered along the cline, we note a cluster of male individuals at the "top" of the cline, suggesting that there is an excess of non-West Eurasian-related ancestry in males relative to females in the Roopkund_A grouping. We next examined the distribution of male and female individuals from the Roopkund_B group on the "West Eurasian" PCA (Supplementary Figure 7b). In this case, we do not observe any qualitative difference in the placement of male and female individuals.

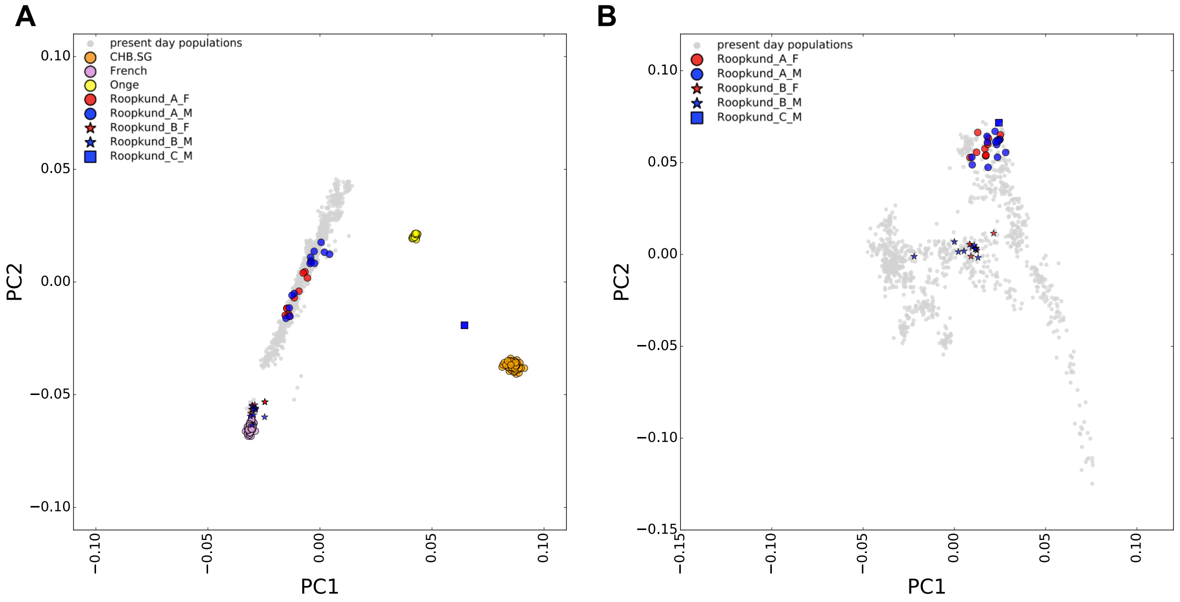

Supplementary Figure 7. Principal Component Analysis of (A) Indian Cline and (B) West Eurasian populations with Roopkund_A and Roopkund_B individuals distinguished by genetic sex. See Figure 2 for full details of each PCA plot.

To test whether there is a significant genetic difference in ancestry between the males and females from each group, we divide the two groups into subpopulations based on sex. We then compute symmetry statistics of the form f_4(Mbuti, *Test*; Male, Female), where *Test* is all populations in the dataset (Supplementary Figure 8, Supplementary Data 19). In the case of the Roopkund_A population, we find numerous instances where the statistic significantly deviates from zero (more than 3 standard deviations), while in the case of Roopkund_B we do not find any significant statistics.

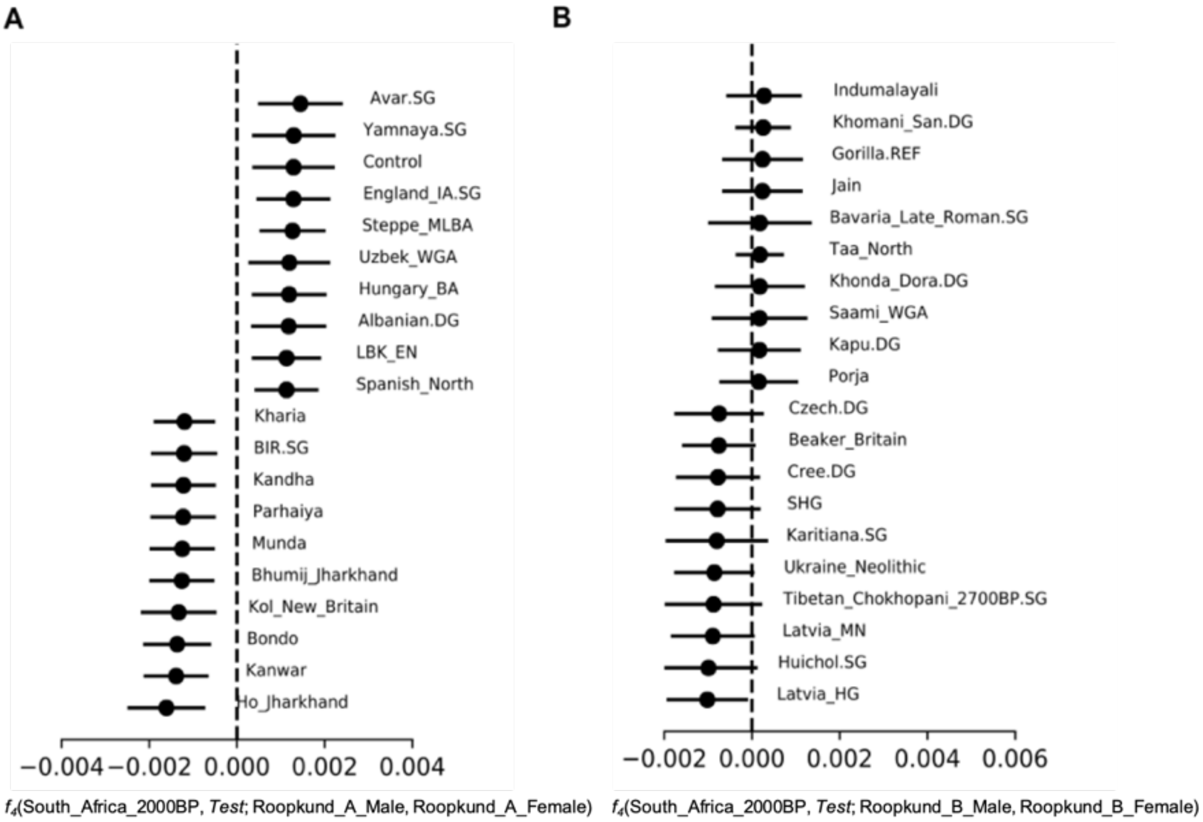

Supplementary Figure 8. f_4-statistics of the form f_4(South_Africa_2000BP, Test; Roopkund_Male, Roopkund_Female), for Roopkund_A (left) and Roopkund_B (right), where Test is all populations in the dataset. The top 10 most positive and most negative statistics are shown (full results are given in Supplementary Data 19). Error bars indicate ±3 standard errors. Source data are provided as a Source Data file.

Although these results suggest that there is a significant difference between the genetic composition of the Roopkund_A males and females, this difference may be an artifact of the random sample of male and female individuals in the dataset. We therefore performed a permutation test to determine whether this result is likely to have been obtained randomly. We randomly assigned each individual in the Roopkund_A population to one of two groups, with group sizes corresponding to the number of males and females in the true groups. When we analyzed the real data, the most significant statistic was observed when Steppe_MLBA was used as the *Test* population. We therefore recomputed this statistic f_4(Mbuti, Steppe_MLBA; Roopkund_A_group1, Roopkund_A_group2) for each simulation, and determined the associated Z-score. We ran this simulation 10,000 times and observe the distribution of z-scores (Supplementary Figure 9). We find that in 150 out of 10,000 cases, the Z-score obtained is higher than the true observed Z-score, corresponding to a randomization-based p-value of 0.015. Thus, there is weakly significant evidence (at p<0.05) but not strongly significant

evidence (at p<0.01) of genetic differences between males and females in the Roopkund_A group.

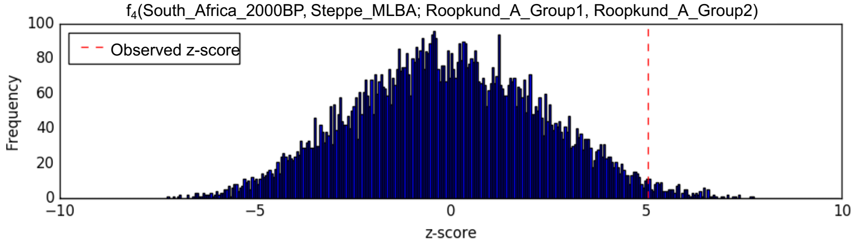

Supplementary Figure 9. The frequency of Z-scores produced by 10,000 permutations of f_4-statistics of the form f_4(South_Africa_2000_BP, Steppe_MLBA; Roopkund_A_Group1, Roopkund_A_Group2). Source data are provided as a Source Data file.

Supplementary Note 9- Constraints on the origin of Roopkund_B

The identification of a group of individuals with eastern Mediterranean-related ancestry in the remote Himalayan site of Roopkund Lake dating to around the 17th century raises the question of when this group came to the region. Although it seems most plausible that individuals in the Roopkund_B group were themselves travelers who were visiting South Asia, we also considered the alternative possibility that Roopkund_B might represent a genetically isolated population with distant eastern Mediterranean ancestry that had been living in the region for many generations.

There are several South Asian groups that identify as Indo-Greek—claiming descent from the army of Alexander the Great. However, these groups (such as the Kalash) possess substantial South Asian-related admixture[32,33]. The Roopkund_B population does not possess any discernable South Asian related ancestry (as evidenced by the genetic analyses described in the main text, particularly *f*-statistic-based analyses, such as *qpAdm*) and is therefore genetically distinct from known groups who claim Indo-Greek ancestry.

If the Roopkund_B individuals did descend from a population with eastern Mediterranean-related ancestry that has been genetically isolated in the Himalayan region for many generations without experiencing any South Asian admixture, we might expect to see genetic signatures of this extended genetic isolation. In order for such a highly isolated group to persist without admixing with local populations, the group would need to have practiced strict endogamy (i.e. always mating with individuals from within their genetic group). Such a practice might be expected to contribute to reduced diversity in an isolated population of small enough size to remain otherwise undetected.

We therefore considered the amount of genetic diversity in the Roopkund_B population by measuring relative levels of conditional heterozygosity using *popstats*[34]. Conditional

heterozygosity is calculated by randomly sampling alleles from two individuals belonging to a single population and calculating the probability of two random sequences mismatching at each site. In populations that are more diverse, the probability of sampling two different alleles at each is greater than in a population that lacks genetic diversity.

Although there are many factors that contribute to the value of conditional heterozygosity measured in a population, we would expect that highly inbred, genetically isolated populations would have relatively lower conditional heterozygosity than populations that are not inbred. We therefore compared the conditional heterozygosity measured in Roopkund_B individuals with the distribution of conditional heterozygosity measured in other ancient European populations dating to within the last 5000 years (Supplementary Data 20). Supplementary Figure 10 shows that the conditional heterozygosity measured in the Roopkund_B population is high relative to the distribution of values observed in other ancient populations with comparable or greater coverage. These findings indicate that the Roopkund_B population is unlikely to be descended from a small, inbred population that has been isolated from its eastern Mediterranean source population for a large number of generations.

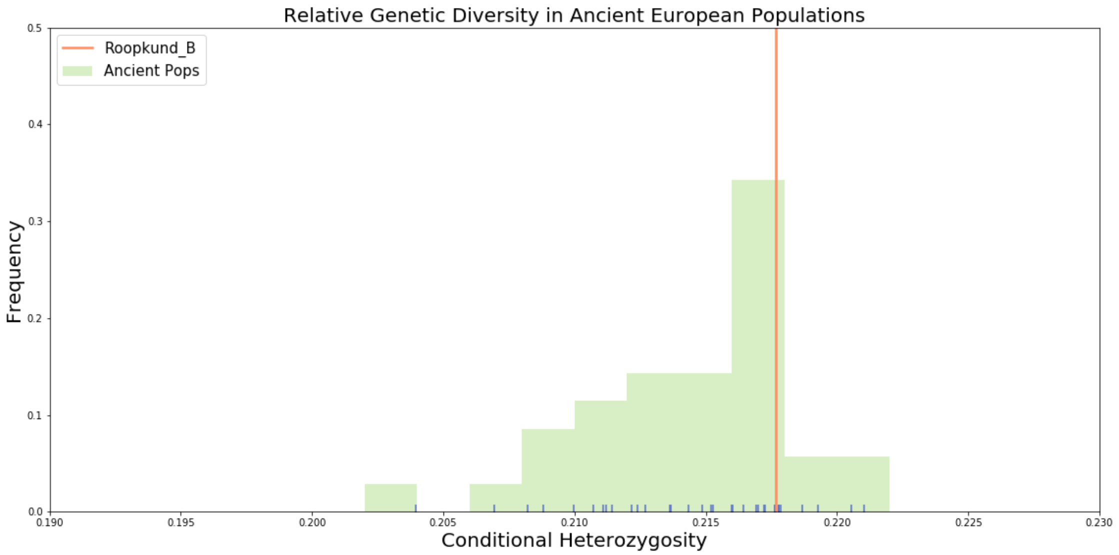

Supplementary Figure 10 Relative Genetic Diversity in Roopkund_B. The distribution of the level of genetic diversity (measured by conditional heterozygosity) in all ancient European populations with a minimum of 20,000 SNPs (shown in light green, exact values for each population are indicated by blue vertical lines at the bottom of the plot). The conditional heterozygosity measured in the Roopkund_B population is indicated by a vertical orange line. Source data are provided as a Source Data file.

A further line of evidence that supports this conclusion is the failure to identify any close relatives (3rd degree of closer) within the Roopkund_B group, as we would expect there to be an increased likelihood of observing close relatives in a small, endogamous population.

The relatively high level of genetic diversity observed in the Roopkund_B population suggests that these individuals do not descend from a small, genetically isolated population that has lived in the Himalayan region for many generations.

References

1. “Skeleton Lake”. *Riddles of the Dead. National Geographic.* Hoggard Films (2004). Television
2. Yang, D. Y., Eng, B., Waye, J. S., Dudar, J. C. & Saunders, S. R. Improved DNA extraction from ancient bones using silica-based spin columns. *Am. J. Phys. Anthropol.* **105**, 539-543 (1998).
3. Herbig, A. *et al.* MALT: Fast alignment and analysis of metagenomic DNA sequence data applied to the Tyrolean Iceman. Preprint at https://www.biorxiv.org/content/10.1101/050559v050551 (2016).
4. Fu, Q. *et al.* DNA analysis of an early modern human from Tianyuan Cave, China. *Proc. Natl. Acad. Sci.* **110**, 2223-2227 (2013).
5. Mathieson, I. *et al.* Genome-wide patterns of selection in 230 ancient Eurasians. *Nature* **528**, 499-512 (2015).
6. Haak, W. *et al.* Massive migration from the steppe was a source for Indo-European languages in Europe. *Nature* **522**, 207-211 (2015).
7. Fu, Q. *et al.* An early modern human from Romania with a recent Neanderthal ancestor. *Nature* **524**, 216-219 (2015).
8. Hübler, R. & Key, F. M. (in prep).
9. Key, F. M., Posth, C., Krause, J., Herbig, A. & Bos, K. I. Mining metagenomic data sets for ancient DNA: recommended protocols for authentication. *Trends Genet.* **33**, 508-520 (2017).
10. Maczulak, A. Clostridium in *Encyclopedia of microbiology*. 168-173 (Facts on File, New York, NY, 2011).
11. Vogel, J. C. & Van Der Merwe, N. J. Isotopic evidence for early maize cultivation in New York State. *Am. Antiq.* **42**, 238-242 (1977).
12. Schoeninger, M. J., DeNiro, M. J. & Tauber, H. Stable nitrogen isotope ratios of bone collagen reflect marine and terrestrial components of prehistoric human diet. *Science* **220**, 1381-1383 (1983).
13. Lee-Thorp, J. A., Sealy, J. C. & Van der Merwe, N. J. Stable carbon isotope ratio differences between bone collagen and bone apatite, and their relationship to diet. *J. Archaeol. Sci.* **16**, 585-599 (1989).
14. Richards, M. P., Schulting, R. J. & Hedges, R. E. Archaeology: sharp shift in diet at onset of Neolithic. *Nature* **425**, 366 (2003).
15. Santana-Sagredo, F., Lee-Thorp, J. A., Schulting, R. & Uribe, M. Isotopic evidence for divergent diets and mobility patterns in the A tacama D esert, northern C hile, during the L ate I ntermediate P eriod (AD 900–1450). *Am. J. Phys. Anthropol.* **156**, 374-387 (2015).
16. DeNiro, M. J. & Epstein, S. Influence of diet on the distribution of nitrogen isotopes in animals. *Geochimica et cosmochimica acta* **45**, 341-351 (1981).
17. Schoeninger, M. J. & DeNiro, M. J. Nitrogen and carbon isotopic composition of bone collagen from marine and terrestrial animals. *Geochim. Cosmochim. Acta* **48**, 625-639 (1984).

18 Ambrose, S. H. & Norr, L. Experimental evidence for the relationship of the carbon isotope ratios of whole diet and dietary protein to those of bone collagen and carbonate in *Prehistoric human bone*. 1-37 (Springer, Berlin, 1993).
19 O'Leary, M. H. Carbon isotope fractionation in plants. *Phytochemistry* **20**, 553-567 (1981).
20 Farquhar, G. D., Ehleringer, J. R. & Hubick, K. T. Carbon isotope discrimination and photosynthesis. *Annu. Rev. Plant Biol.* **40**, 503-537 (1989).
21 Smith, B. N. & Epstein, S. Two categories of 13C/12C ratios for higher plants. *Plant Physiol.* **47**, 380-384 (1971).
22 Hedges, R. E. & Reynard, L. M. Nitrogen isotopes and the trophic level of humans in archaeology. *J. Archaeol. Sci.* **34**, 1240-1251 (2007).
23 Heaton, T. H. The 15 N/14 N ratios of plants in South Africa and Namibia: relationship to climate and coastal/saline environments. *Oecologia* **74**, 236-246 (1987).
24 Fogel, M. L., Tuross, N., Johnson, B. J. & Miller, G. H. Biogeochemical record of ancient humans. *Org. Geochem.* **27**, 275-287 (1997).
25 Bogaard, A., Heaton, T. H., Poulton, P. & Merbach, I. The impact of manuring on nitrogen isotope ratios in cereals: archaeological implications for reconstruction of diet and crop management practices. *J. Archaeol. Sci.* **34**, 335-343 (2007).
26 Minagawa, M. & Wada, E. Stepwise enrichment of 15N along food chains: further evidence and the relation between δ15N and animal age. *Geochim. Cosmochim. Acta* **48**, 1135-1140 (1984).
27 Moorjani, P. *et al.* Genetic evidence for recent population mixture in India. *Am. J. Hum. Genet.* **93**, 422-438 (2013).
28 Reich, D. *et al.* Reconstructing native American population history. *Nature* **488**, 370-374 (2012).
29 Black, M. L., Dufall, K., Wise, C., Sullivan, S. & Bittles, A. H. Genetic ancestries in northwest Cambodia. *Ann. Hum. Biol.* **33**, 620-627 (2006).
30 Patterson, N., Price, A. L. & Reich, D. Population structure and eigenanalysis. *PLoS Genet.* **2**, e190 (2006).
31 Nakatsuka, N. *et al.* The promise of discovering population-specific disease-associated genes in South Asia. *Nature Genet.* **49**, 1403-1407 (2017).
32 Hellenthal, G. *et al.* A genetic atlas of human admixture history. *Science* **343**, 747-751 (2014).
33 Ayub, Q. *et al.* The Kalash genetic isolate: ancient divergence, drift, and selection. *Am. J. Hum. Genet.* **96**, 775-783 (2015).
34 Skoglund, P. *et al.* Genetic evidence for two founding populations of the Americas. *Nature* **525**, 104 (2015).
