# Supplementary material for: Ancient DNA from the skeletons of Roopkund Lake reveals Mediterranean migrants in India
Source: Nat Commun. 2019 Aug 20;10:3670. doi: 10.1038/s41467-019-11357-9 (PMC6702210; doi:10.1038/s41467-019-11357-9)
Supplement: Supplementary file 3 — Reporting Summary [file 41467_2019_11357_MOESM3_ESM.pdf]

nature research

Corresponding author(s): Eadaoin Harney, Ayushi Nayak, David Reich, Niraj Rai

Last updated by author(s): Jun 5, 2019

Reporting Summary

Nature Research wishes to improve the reproducibility of the work that we publish. This form provides structure for consistency and transparency in reporting. For further information on Nature Research policies, see Authors & Referees and the Editorial Policy Checklist.

Statistics

For all statistical analyses, confirm that the following items are present in the figure legend, table legend, main text, or Methods section.

n/a	Confirmed	
☐	☒	The exact sample size (*n*) for each experimental group/condition, given as a discrete number and unit of measurement
☐	☒	A statement on whether measurements were taken from distinct samples or whether the same sample was measured repeatedly
☐	☒	The statistical test(s) used AND whether they are one- or two-sided<br>*Only common tests should be described solely by name; describe more complex techniques in the Methods section.*
☒	☐	A description of all covariates tested
☐	☒	A description of any assumptions or corrections, such as tests of normality and adjustment for multiple comparisons
☐	☒	A full description of the statistical parameters including central tendency (e.g. means) or other basic estimates (e.g. regression coefficient) AND variation (e.g. standard deviation) or associated estimates of uncertainty (e.g. confidence intervals)
☐	☒	For null hypothesis testing, the test statistic (e.g. *F*, *t*, *r*) with confidence intervals, effect sizes, degrees of freedom and *P* value noted<br>*Give P values as exact values whenever suitable.*
☐	☒	For Bayesian analysis, information on the choice of priors and Markov chain Monte Carlo settings
☐	☒	For hierarchical and complex designs, identification of the appropriate level for tests and full reporting of outcomes
☒	☐	Estimates of effect sizes (e.g. Cohen's *d*, Pearson's *r*), indicating how they were calculated

Our web collection on statistics for biologists contains articles on many of the points above.

Software and code

Policy information about availability of computer code

Data collection	No software was used in the collection of data.
Data analysis	We used published population genetics software tools: qpDstat (v711), qp3Pop (v420), qpWave (v410), qpAdm (v810), Haplogrep2, smartpca (v16251), ADMIXTURE (v1.23), Plink (v1.07), OxCal (v4.3.2), Bwa (v0.6.1), ANGSD (v0.911), popstats

For manuscripts utilizing custom algorithms or software that are central to the research but not yet described in published literature, software must be made available to editors/reviewers. We strongly encourage code deposition in a community repository (e.g. GitHub). See the Nature Research guidelines for submitting code & software for further information.

Data

Policy information about availability of data

All manuscripts must include a data availability statement. This statement should provide the following information, where applicable:

- Accession codes, unique identifiers, or web links for publicly available datasets
- A list of figures that have associated raw data
- A description of any restrictions on data availability

The aligned DNA sequences from the 38 individuals are available from the European Nucleotide Archive under accession number PRJEB29537 [https://www.ebi.ac.uk/ena/data/view/ PRJEB29537]. Genotype files are available at https://reich.hms.harvard.edu/datasets. All other relevant data is available upon request.

Field-specific reporting

Please select the one below that is the best fit for your research. If you are not sure, read the appropriate sections before making your selection.

☒ Life sciences ☐ Behavioural & social sciences ☐ Ecological, evolutionary & environmental sciences

For a reference copy of the document with all sections, see nature.com/documents/nr-reporting-summary-flat.pdf

Life sciences study design

All studies must disclose on these points even when the disclosure is negative.

Sample size	Sample sizes were not predetermined; as many ancient samples as possible were included in the analyses.
Data exclusions	As described in the text, some samples were excluded from analysis based on poor data quality.
Replication	Mitochondrial haplogroups were determined using two independent processes, one PCR-based method performed at the CCMB in Hyderabad, India, and another capture-based method performed at Harvard Medical School in Boston, USA. Results appear to be generally consistent, with minor differences in calls most likely attributable to differences in sequencing coverage/processing methods. Stable isotope data was obtained for one sample in two separate laboratories, and values were consistent between the two labs.
Randomization	No randomization was performed. We grouped samples based on radiocarbon dates and genetic ancestry.
Blinding	The investigators were not blinded to group allocation during data collection and analysis

Reporting for specific materials, systems and methods

We require information from authors about some types of materials, experimental systems and methods used in many studies. Here, indicate whether each material, system or method listed is relevant to your study. If you are not sure if a list item applies to your research, read the appropriate section before selecting a response.

Materials & experimental systems

n/a	Involved in the study	
☒	☐	Antibodies
☒	☐	Eukaryotic cell lines
☒	☐	Palaeontology
☒	☐	Animals and other organisms
☐	☒	Human research participants
☒	☐	Clinical data

Methods

n/a	Involved in the study	
☒	☐	ChIP-seq
☒	☐	Flow cytometry
☒	☐	MRI-based neuroimaging

Human research participants

Policy information about studies involving human research participants

Population characteristics	We generated genome-wide data from 104 individuals, belonging to 5 populations from the Indian Himalayan region neighboring Roopkund Lake: Ladakh (n=88), Dewal (n=5), Baimaru (n=5), Wan (n=3), and Tiirpak (n=3). Donors ranged in age from 10-50 years old, and are predominantly male. This data is described in Supplementary Information section 7.
Recruitment	Samples were collected from unrelated individuals living in villages surrounding the Roopkund Lake area on a volunteer basis, with their informed written consent.
Ethics oversight	Institutional Ethical Committee of the Centre for Cellular and Molecular Biology in Hyderabad, India.

Note that full information on the approval of the study protocol must also be provided in the manuscript.
