# Supplementary material for: Ancient DNA from the skeletons of Roopkund Lake reveals Mediterranean migrants in India
Source: Nat Commun. 2019 Aug 20;10:3670. doi: 10.1038/s41467-019-11357-9 (PMC6702210; doi:10.1038/s41467-019-11357-9)
Supplement: Supplementary file 4 — Description of Additional Supplementary Files [file 41467_2019_11357_MOESM4_ESM.pdf]

Description of Additional Supplementary Files

- **Supplementary Data 1**. Summary of data for each library
- **Supplementary Data 2.** Pairwise F_{ST} values used in Figure 2c,d
- **Supplementary Data 3.** Identifying Potential Outliers Using f_4-statistics of the form f_4Roopkund_B_exclude_target, target; Test, Chimp)
- **Supplementary Data 4.** Isotopic Measurements
- **Supplementary Data 5.** Modeling Roopkund_B as a Clade with Present-Day Populations
- **Supplementary Data 6.** Y chromosome haplogroup determination
- **Supplementary Data 7.** Diagnostic mitochondrial sites by pool
- **Supplementary Data 8.** Mitochondrial Genotyping Raw Data
- **Supplementary Data 9**. f_4(Roopkund_B, South_Africa_2000BP; Crete.DG, Test)
- **Supplementary Data 10.** f_4(South_Africa_2000BP, Test; Crete.DG, Roopkund_B)
- **Supplementary Data 11**. f_3(Roopkund_B; Crete.DG, Test)
- **Supplementary Data 12.** Modeling Individuals from Roopkund_A as a clade with present-day populations
- **Supplementary Data 13.** Modeling Roopkund_A individuals as 2-way admixture of two present-day populations
- **Supplementary Data 14**. Estimates of West Eurasian-related ancestry proportion in Roopkund_A individuals via statistics of the form f_4(Yoruba,Basque;Test,Onge)/ f_4(Yoruba,Basque;Georgian,Onge)
- **Supplementary Data 15.** Modeling Roopkund_C as a Clade with Present-Day Populations
- **Supplementary Data 16.** Modeling Roopkund_C as 2-way admixture of two present-day populations
- **Supplementary Data 17.** Modeling Roopkund_C as 2-way admixture of two present-day populations including Cambodian.DG
- **Supplementary Data 18.** Pairwise F_{ST} with present-day Himalayan Populations
- **Supplementary Data 19.** Testing for Assymetry in Genetic Affinities Between Sexes Using symmetry f_4-statistics of the form f_4(South_Africa_2000BP,Test;Roopkund_X_M, Roopkund_X_F)
- **Supplementary Data 20.** Conditional Heterozygosity
