# Supplementary material for: Ancient DNA from the skeletons of Roopkund Lake reveals Mediterranean migrants in India
Source: Nat Commun. 2019 Aug 20;10:3670. doi: 10.1038/s41467-019-11357-9 (PMC6702210; doi:10.1038/s41467-019-11357-9)
Supplement: Supplementary file 2 — Peer Review File [file 41467_2019_11357_MOESM2_ESM.pdf]

Editorial Note: This manuscript has been previously reviewed at another journal that is not operating a transparent peer review scheme. This document only contains reviewer comments and rebuttal letters for versions considered at Nature Communications .

REVIEWERS' COMMENTS:

Reviewer #1 (Remarks to the Author):

I think the authors have answered my previous questions and also improved the clarity of the manuscript in some sections; it is still a fascinating and intriguing story that deserves to be published at Nat Comm and that will likely draw lots of attention.

Reviewer #2 (Remarks to the Author):

n/a

REVIEWERS' COMMENTS:

- Reviewer #1 (Remarks to the Author):

I think the authors have answered my previous questions and also improved the clarity of the manuscript in some sections; it is still a fascinating and intriguing story that deserves to be published at Nat Comm and that will likely draw lots of attention.

- Reviewer #2 (Remarks to the Author):

n/a

We thank all of the reviewers for their comments and valuable critiques, which have improved the manuscript.
